# Characterization of *TaDREB1* in wheat genotypes with different seed germination under osmotic stress

**DOI:** 10.1186/s41065-018-0064-6

**Published:** 2018-08-01

**Authors:** Meng Liu, Zeng Wang, Hong-mei Xiao, Yan Yang

**Affiliations:** College of Life Sciences, Inner Mongolia Key Laboratory of Plant Stress Physiology and Molecular Biology, Inner Mongolia Agricultural University, Erdos Road, Hohhot, 010018 Inner Mongolia China

**Keywords:** Nucleotide polymorphism, Expression characteristics, *TaDREB1*, Osmotic stress resistance

## Abstract

**Background:**

The cis-acting element DRE/CRT plays an important role in activating gene expression responsive to osmotic stress, low temperature and high-salinity. *DREB1*/*CBF* genes encode DRE-binding proteins with the function as transcript activators. *TaDREB1* was also found to be induced by osmotic stress.

**Methods:**

The dates of osmotic stress was assessed by seed germination drought resistance index; the full-length cDNA sequences of *TaDREB1* gene were downloaded from NCBI datebase; identification of allelic variation and transcript expression were assessed by PCR and semi-quantitive RT-PCR analysis, respectively.

**Results:**

Total 13 new allele variations of ***TaDREB1*** were identified in the germplasms tested in the paper, including 5 ***TaDREB1****-A* on chromosome 3AL, 4 ***TaDREB1****-B* on chromosome 3BL and 4 ***TaDREB1****-D* on chromosome 3DL. In each variety, there existed two loci of *TaDREB1-D* genes, named *TaDREB1-D1* and *TaDREB1-D2*, both of which had the similar nucleotide sequence except an 11 bp insertion in the former. In wheat seeds under osmotic stress, we did not detect the transcript expression level of *TaDREB1-A* and *TaDREB1-B*, but that of *TaDREB1-D*.

**Conclusions:**

The capacity of osmotic stress tolerance was closely correlated with the expression level and tendency of *TaDREB1-D*.

**Electronic supplementary material:**

The online version of this article (10.1186/s41065-018-0064-6) contains supplementary material, which is available to authorized users.

## Background

Drought is already widespread in many regions and one of the primary reason causing plant loss worldwide. [1]. Even in many irrigated regions, shortage of water supply allows only limited irrigation. Therefore, it is imperative to strengthen the study on osmotic stress resistance of wheat.

Five multi-gene families belong to transcript factors [bZIP (mainly AREB/ABF), DREB (AP2/EREBP), MYB/MYC, NAC and WRKY)] have been reported to be associated with drought tolerance. A number of wheat genes for transcript factors (*TabZIP1*, *TabZIP60*, *TaABRE3*, *TaDREB1*, *TaPIMP1*, *TaNAC29* and *TAWRKY44*) have been shown to exhibit the induced expression during exposure to drought stress, suggesting that these genes may be used for improving stress tolerance in wheat [2–9].

Dehydration-responsive element binding (DREB) proteins constitute a large family of transcription factors regulating some functional genes closely related to drought, high-salinity and low temperature [10, 11]. *Arabidopsis* genome has six *DREB1*/*CBF* genes, namely *DREB1A*/*CBF3*, *DREB1B*/*CBF1*, *DREB1C*/*CBF2*, *DREB1D*/*CBF4*, *DDF1*/*DREB1F* and *DDF2*/*DREB1E* [12]. *DREB* genes feature three conserved regions, an EREBP/AP2 DNA binding domain [13, 14], an N-terminal nuclear localization signal, and a conserved Ser/Thr-rich region adjacent to the EREBP/AP2 domain [15, 16]. The characteristics of above three regions determine the *DREB* characteristics [17]. Most of the functional studies on *DREB* transcription factors were focused on *Arabidopsis thaliana* before 2002, and then in wheat, rice, soybean, cotton and other plants [18–24]. *DREB* factors have been extensively characterized and grouped into several molecular classes based on similarities in the function or the amino acid sequence of the proteins they encode [24]. *DREB*1 and *DREB2* are two main subgroups of the *DREB* subfamily, involved in two different signal transduction pathways under cold and dehydration, respectively [25]. *DREB1* genes include *DREB1-A*, *DREB1-B*, *DREB1-C*,*DREB1-D*,*DREB1-E* and *DREB1F* [26]*. TaDREB1* genes were located on chromosomes 3A, 3B and 3D [11], and isolated from a drought-induced cDNA library of wheat, which was found to be induced by low temperature, abscisic acid (ABA), salinity and drought [27]. The cDNA length of *TaDREB*1 is 1292 bp, including 837 bp open reading frame, 251 bp 5’-UTR and 204 bp 3’-UTR [18]. The study results showed that the heterogeneity of *TaDREB1* gene haplotypes is inconsistent in drought-resistant materials or in drought-sensitive wheat materials, indicating the complexity of drought resistance. In addition, the nucleic acid polymorphisms of *TaDREB1* gene are richer in wheat [26].

The present research aimed at identifying the seed germination resistance varieties under osmotic stress for wheat breeding program, searching the new allelic variation of *TaDREB1*, looking for SNPs, InDels and transcript expression level associated with seed germination tolerance under osmotic stress, and charactering the transcript expression level of *TaDREB1-A*, *TaDREB1-B* and *TaDREB1-D* in wheat varieties or lines with different values of GDRI (germination drought resistance index).

## Methods

### Plant materials

Six spring wheat genotypes were used for cloning of *TaDREB1-A*, *TaDREB1-B* and *TaDREB1-D* genes, including three osmotic stress-resistant genotypes (08–1783, 08–1826 and 07–6239 with relative germination rate values of 55.98, 47.74 and 47.11%, respectively) and three osmotic stress-susceptible ones (08dongzhong 275, Zhangye 1 and 05 cm 178 with relative germination rate values of 1.27, 0.51 and 0%, respectively). Eighty Chinese spring wheat genotypes, with different osmotic stress tolerance from China Spring-sown Wheat Region (CSWR) were used for identifying the osmotic stress tolerance (Table 1). Mature seeds of 08–1783 and Zhangye 1 were used to extract RNA for analysis of mRNA transcription.

**Table 1:** Primer sequence used in this study

Primer	Primer sequence(5′-3′)	Fragment size (bp)	Anneal temp.(°C)
DREB1-A1F	CGGAACCACTCCCTCCATCTC	1107	62
DREB1-A1R	CGGTTGCCCCATTAGACGTCA		
DREB1-A2F	CTGGCACCTCCATTGCTGAC	599	63
DREB1-A2R	AGTACATGAACTCAACGCACAGGACAAC		
DREB1-BF	CCCAACCCAAGTGATAATAATCT	716	58
DREB1-BR	TTGTGCTCCTCATGGGTACTT		
DREB1-D1F	TCGTCCCTCTTCTCGCTCCAT	1190	63
DREB1-D1R	GCGGTTGCCCCATTAGACATCG		
DREB1-D2F	CTGGCACCTCCATTGCCGAT	596	64
DREB1-D2R	AGTACATGAACTCAACGCACAGGACAAC		
DREB U	TCGTCCCTCTTCTCGCTCCATGG	493	66
DREB D	GGGCATGGCG CCGCATGG		
DREB1-AF	ATGAACAGGAAGAAGAAAGTGCGC	593	62
DREB1-AR	TTCTCAAATCATTGCTCACT TCTTTC		
DREB1-BF	ATGACCAGGAAGAAGAAAGTGCGC	585	60
DREB1-BR	TCATTGCTCACTTCTTTTTTCACCTTAT		
DREB1-DF	ATGAACAGGAAGAAGAAAGTGCGC	455	62
DREB1-DR	TCCTTCCCATCAGAAGGATGTGAC		
β-actin F	GTTTCCTGGAATTGCTGATCGCAT	410	65
β-actin R	CATTATTTCATACAGCAGGCAAGC		

### Methods

#### Assay for seed germination drought tolerance

Osmotic stress was assessed based on seed germination drought resistance index (GDRI). Ears were harvested at dough-yellow ripening stage, hand-threshed and sterilized with HgCl_2_, and then placed in plastic petri dishes on filter paper with 6 mL of treatment solution (− 1.00 MPa) at room temperature, and distilled water was used as a control. Germinated seeds were counted and removed daily. The number of germinated seeds was investigated on the 2nd, 4th, 6th and 8th days (seeds with over 1 mm length of radicle were counted). The GDRI values was calculated according to the following formula:

$$ {GDRI}_i=\frac{GIsi}{GIci}\times 100\% $$

$$ GDRI=\sum \limits_{i=1}^3\mathrm{GDRI}i/3 $$

$$ GI=\left(1.00\mathrm{nd}2+\kern0.37em 0.75\mathrm{nd}4+\kern0.37em 0.50\mathrm{nd}6+\kern0.37em 0.25\mathrm{nd}8\right)/N\times \kern0.37em 100 $$

*GDRI-*germination drought resistance index of seeds per reparation.

*GIs*-germination index of seeds under stress.

*GIc-*germination index of seeds as control.

nd2, nd4, nd6, nd8 -represents the number of seeds germinated on day 2, 4, 6 and 8, respectively.

N-represents the total number of seeds.

Then using the membership function method in fuzzy mathematic, the average germination index of wheat under osmotic stress was calculated to evaluate the difference of osmotic stress resistance among the varieties [28, 29].

1 $$ \mathrm{U}\left({\mathrm{x}}_{\mathrm{j}}\right)=\frac{\mathrm{x}\mathrm{j}-\mathrm{xjmin}}{\mathrm{x}\mathrm{j}\mathrm{max}-\mathrm{xjmin}\mathrm{j}}=1,\kern0.5em 2,\kern0.5em 3 $$

2 $$ \mathrm{U}\left({\mathrm{x}}_{\mathrm{j}}\right)=1-\frac{\mathrm{x}\mathrm{j}-\mathrm{xjmin}}{\mathrm{x}\mathrm{j}\mathrm{max}-\mathrm{xjmin}\mathrm{j}}=1,2,3 $$

3 $$ \mathrm{D}={\sum}_{j=1}^n\left[\mathrm{U}\left(\mathrm{xj}\right)\frac{\left|\mathrm{rj}\right|}{\sum_{j=1}^n\left|\mathrm{rj}\right|}\right]=1,2,3 $$

X_j_----the jth measured value of osmotic stress resistance index;

X_j_max-----the maximum value of the jth osmotic stress resistance index.

X_j_min------the minimum value of the jth osmotic stress resistance index.

U(x_j_)--the membership function value of the jth index.

Rj-----The correlation coefficient between the jth index and the comprehensive 106 osmotic stress resistance coefficient.

*R*_*j*_/ ∑ *nj* = *1rj* ------As the indicator weight, indicating the importance of the jth index in all indicators.

D---The evaluated value of each osmotic stress resistance indicator for each species under mannitol stress.

If the measured index is positively correlated with the osmotic stress resistance of the genotypes, then formula (1) is used to calculate the membership function value, otherwise (2). Using D values to evaluate the osmotic stress resistance of the species, that is, D > 0.80 is for Level 1 (strong resistance), 0.50 < D < 0.80 is for Level 2 (resistance), 0.30 < D < 0.5 is for Level 3 (weak resistance), D < 0.30 is for Level 4 (sensitive). The entire osmotic stress resistance index was treated with Excel system.

### Primer design

Based on the full-length cDNA sequence of *TaDREB1* gene(AF303376), the primers were designed using DNAMAN software and were synthesized in Takara Biotechnology (Dalian) Co. Ltd. in China (Table 2).

**Table 2:** Values of GDRI in 80 spring wheat varieties and lines

No	Varieties and lines	GDRI (2015)%	GDRI (2016)%	Average GDRI (%)	D values
1	08–1783	22.57	89.39	55.98	1.00
2	07–5866	22.77	74.18	48.47	0.87
3	08–1826	29.71	65.78	47.74	0.85
4	07–6239	21.66	72.57	47.11	0.84
5	Bayou 2	22.83	68.27	45.55	0.81
6	34–206	6.74	83.69	45.22	0.81
7	Linyou 2	22.95	66.17	44.56	0.80
8	08–1718	9.16	77.47	43.31	0.77
9	08–2294	9.51	75.42	42.46	0.76
10	08–3348	7.8	76.92	42.36	0.76
11	Jinsui0095	1.66	80.11	40.88	0.73
12	Qingchun37	6.07	75.46	40.77	0.73
13	Bayou1	7.38	73.62	40.5	0.72
14	Linyou 1	10.99	68.57	39.78	0.71
15	73B609	13.47	63.03	38.25	0.68
16	8916–40	12.94	63.41	38.17	0.68
17	Bi 1ama0	12.39	62.47	37.43	0.67
18	Wu E32–1	13.01	61.71	37.36	0.67
19	Yong2739	0	70.26	35.13	0.63
20	Jiu9996	2.08	63.58	32.83	0.59
21	Jinsui8145	7.57	50.14	28.86	0.52
22	08dongzhong2455	7.26	48.33	27.8	0.50
23	Qingchun556	12.07	43.37	27.72	0.50
24	05 cm220	0.75	53.45	27.1	0.48
25	05–1173	3.92	46.72	25.32	0.45
26	Lafan8	8.97	39.75	24.36	0.44
27	95y16	11.41	32.34	21.88	0.39
28	Zhongning31051	8.62	32.69	20.65	0.37
29	Ganchun20	11.54	26.67	19.1	0.34
30	Ningchun10	4.35	32.09	18.22	0.33
31	Bafeng1	16.78	18.43	17.6	0.31
32	NingPS184	1.01	33.76	17.39	0.31
33	La2676–9	1.92	32.84	17.38	0.31
34	Yemao	16.34	18.18	17.26	0.31
35	G47	16.92	15.74	16.33	0.29
36	Zhongguochun	9.29	22.43	15.86	0.28
37	C1845	5.73	24.5	15.12	0.27
38	08dongzhong6741	4.55	25.12	14.83	0.26
39	Jiaoyuan356	16.47	10.92	13.7	0.24
40	Xiaobing32	4.12	22.59	13.35	0.24
41	08–1699	0.64	25.93	13.28	0.24
42	08dongzhong1791	11.54	14.22	12.88	0.23
43	07–6228	3.03	22.19	12.61	0.23
44	Ganchun22	8	16.77	12.39	0.22
45	Bafeng3	3.17	19.93	11.55	0.21
46	03 cm-338	0	22.46	11.23	0.20
47	Qinghai 932	0	22.37	11.18	0.20
48	08dongzhong1807	1.76	18.31	10.03	0.18
49	05–5371	0.52	17.03	8.77	0.16
50	Luobupin	16.19	0.88	8.54	0.15
51	Wu M27–2	0	16.67	8.33	0.15
52	YK6–325	1.45	15.15	8.3	0.15
53	08–353	6.32	9.89	8.11	0.14
54	Bafeng6	8.58	7.37	7.98	0.14
55	Gelanni	1.96	13.91	7.93	0.14
56	82,170–1	8.7	6.67	7.68	0.14
57	Wu M437	3.57	11.76	7.67	0.14
58	Yong 2352	6.11	8.78	7.45	0.13
59	08dongzhong2417	8.8	5.28	7.04	0.13
60	08dongzhong3008	10.71	2.42	6.56	0.12
61	Ningzi08A751	0	12.79	6.39	0.11
62	08dongzhong550	3.21	8.75	5.98	0.11
63	y31	2.26	9.67	5.96	0.11
64	Moyin45	4.76	7.03	5.9	0.11
65	08dongzhong1146	7.69	3.96	5.83	0.10
66	Lanyou5074	0.39	8.67	4.53	0.08
67	08–3410	1.37	6.25	3.81	0.07
68	08H415	3.23	4.38	3.8	0.07
69	07–6751	2.3	4.76	3.53	0.06
70	Hetao3	0	6.97	3.48	0.06
71	Zhangchun11	0	6.25	3.13	0.06
72	Yong 2356	0	5.69	2.84	0.05
73	08H274	0.34	3.56	1.95	0.03
74	Jiusandasui7788	2.63	1.23	1.93	0.03
75	Shentai1	0.96	2.2	1.58	0.03
76	08dongzhong275	0	2.53	1.27	0.02
77	08dongzhong1809	0	2.5	1.25	0.02
78	Ningchun35	2.33	0	1.16	0.02
79	Zhangye1	1.02	0	0.51	0.01
80	05 cm178	0	0	0	0.00

### PCR amplification and semi-quantitive RT-PCR analysis

For each varieties, 3 ~ 5 seeds were selected and cultured in a clean culture dish at 25 °C for 5 ~ 7 d. 1 ~ 2 g leaves were frozen by liquid nitrogen. Then genomic DNA was extracted by method of CTAB. The *TaDREB1-A*, *TaDREB1-B* and *TaDREB1-D* were amplified by specific primers (Table 1) in genotypes with different osmotic stress tolerance.

PCR reaction was performed in a PTC-100 TMProgrammable Thermal Controller in a total volume of 15 μL, including 30 ng genomic DNA, 10 × PCR reaction Mix buffer 1.5 μL, 0.3 μM of each primer, 0.5 U of Taq DNA polymerase (TaKaRaCo., China). It was carried out using the following programs: initial denaturation at 94 °C for 5 min; 35 cycles of 72 °C for 30–90s, annealing at 58 °C~ 64 °C for 30 ~ 90s, 72 °C for 30 ~ 90s and a final extension at 72 °C for 10 min. The PCR products recovered by agarose gel were cloned into the cloning vector PMD19-T (TaKaRaCo., China). The sequencing was performed by Nanjing Kingsley Sequencing Co., Ltd. Sequencing data analysis was performed using DNAMAN software (https://www.lynnon.com/pc/framepc.html).

Total RNA was extracted from the mature dry seeds treated with − 1.00 MPa mannitol for 0 h, 12 h, 24 h and 36 h, using TaKaRa Mini BEST plant RNA extraction kit (TaKaRa Co., China). The concentration and purity of the total RNA extracted was determined using a NanoDropND-2000C spectrophotometer. According to the manuscript, cDNA was synthesized with the reverse transcriptase kit RTaseM-MLV (TaKaRa Co., China). RT-PCR reaction was performed in a PTC-100 TMProgrammable Thermal Controller with a total volume of 15 μL, including 30 ng template cDNA, 1.5 μL10 × PCR reaction mix buffer, 0.3 μM of each primer, 0.5 U of Taq DNA polymerase(TaKaRaCo., China). PCR amplification were 94 °C for 5 min; 35 cycles of 72 °C for 30s, annealing at 62 °C for 30s, 72 °C for 30s and a final extension at 72 °C for 10 min. The PCR products were detected by 1% agarose gel.

## Results

### Identification of GDRI in 80 varieties and lines

**Fig. 1:**
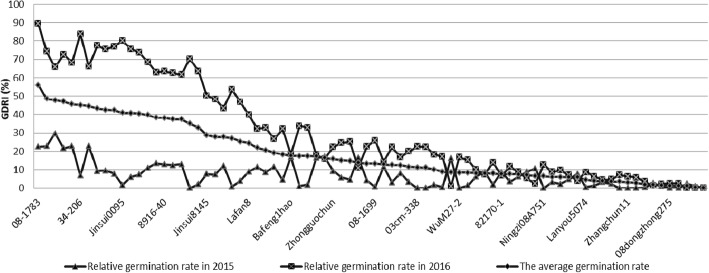
The trend of the seed germination osmotic stress index in 80 spring wheat varieties and lines

The osmotic stress resistance during germination period of wheat seeds was tested by seed germination drought resistance index (GDRI). The GDRI values of the 80 genotypes showed consistence over the 2 years (*R* = 0.467, *P* = 0.000), with mean values and standard deviations were 6.99 ± 6.82 and 30.47 ± 26.84 in the year of 2015 and 2016, respectively (Table 1, Fig. 1).

**Fig. 2:**
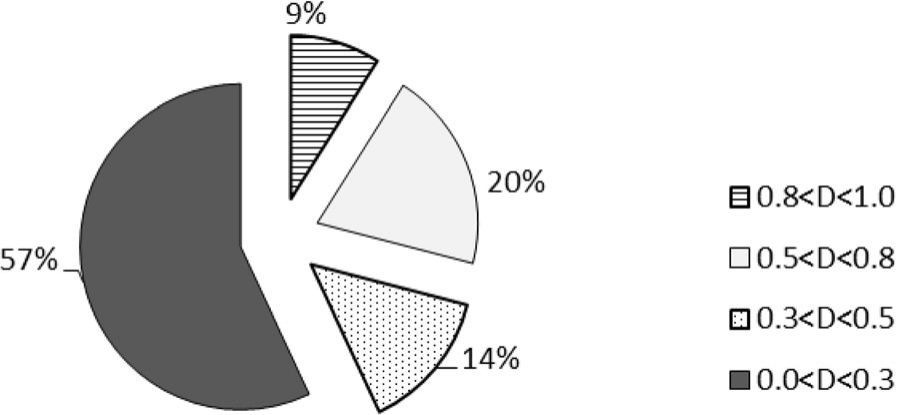
Distribution pattern of seed germination osmotic stress resistance of 80 spring wheat varieties and lines. Note: $D={\sum}_{j=1}^n\left[U(xj)\frac{\left| rj\right|}{\sum_{j=1}^n\left| rj\right|}\right]j=1$, 2, 3 (3), D value means the evaluated value of each drought resistance indicator for each species under mannitol stress; 0.8 < D < 1.0 is strong resistance, 0.8 > D > 0.5 is resistance, 0.5 > D > 0.3 is weak resistance, 0.3 > D > 0 is sensitive.

The degree of osmotic stress resistance during seed germination period was evaluated by the D values of membership function method in fuzzy mathematics. The analysis of results showed that seven genotypes (08–1783, 07–5866, 08–1826, 07–6239, Bayou 2, 34–206 and Linyou 2) belong to the level 1 osmotic stress tolerance (0.8 < D < 1), accounting for 8.75%; Sixteen belong to the level 2 osmotic stress tolerance (0.5 < D < 0.8), accounting for 20.00%; Fifteen belong to the level 3 osmotic stress tolerance (0.5 < D < 0.3), according for 18.75%; And another 46 ones belong to the level 4 osmotic stress tolerance (sensitive, 0 < D < 0.3), according for 57.50% (Fig. 2).

### Identification of allelic variation of *TaDREB1* gene

The specific primers of *DREB1-A1, DREB1-A2, DREB1-B, DREB1-D1* and *DREB1-D2* were used to amplify the *TaDREB1-A1*, *TaDREB1-B1* and *TaDREB1-D1*, respectively, in six genotypes (including three osmotic stress-resistant materials and three osmotic stress-sensitive ones). Total 13 new allele variations of *TaDREB1-A1*, *TaDREB1-B1* and *TaDREB1-D1* were found and named according to the 2005 Supplement of the Wheat Gene Catalogue [30] in the study, i.e. *TaDREB1-A11*, *TaDREB1-A12*, *TaDREB1-A13*, *TaDREB1-A14* and *TaDREB1-A15* on chromosome 2AL; *TaDREB1-B11*, *TaDREB1-B12*, *TaDREB1-B13* and *TaDREB1-B14* on chromosome 2BL; *TaDREB1-D11*, *TaDREB1-D12*, *TaDREB1-D21* and *TaDREB1-D22* on chromosome 2DL.

Compared with *TaDREB1-A* (GenBank accession number: DQ195070.1), 38 SNPs were found in the full sequence of *TaDREB1-A1*, the frequency of SNPs being 2.27%. Among these, 4 SNPs were observed in 5’UTR, 7 SNPs located in the first exons, 23 SNPs found in introns, and the other SNPs were found in the second exons of all alleles of *TaDREB1-A1* except *TaDREB1-A14*. The mutation in 939 bp site is non-synonymous from hydrophobic weak glycine (G) to hydrophobic strong aspartic acid (D), while the mutations in other 3 sites are synonymous. Three base deletions were detected, among which 1 single base T is missed at position 40 bp in 5’UTR of all *TaDREB1-A1* variations, and other 2 bp TT deletions occurred at position 453 bp, in introns of all *TaDREB1-A1* variations except *TaDREB1-A14*. 3 insertions were found at position 540 bp (T), 608 bp (CTG), 629 bp (CT) in introns of all *TaDREB1-A1* variations except *TaDREB1-A14* (Additional file 1: Figure S1).

33SNPs were found in the full sequence of *TaDREB1-B1*, the frequency of SNPs was 4.78%. Among them, 6 SNPs were found in 5’UTR, twenty-seven SNPs were located in exons of *TaDREB1-B11* and *TaDREB1-B12*. One T is inserted at 485 bp site of coding region. T-A, E-D, G-R, T-I, A-P, K-N, E-R, V-I, S-E, N-Q changes were found in the protein sequences. Most of the amino acid mutations were detected in *TaDREB1-A13* genotypes, and the hydrophilicity is enhanced after the amino acid mutation. Lysine (k) is missed. The changes were not consistent with the osmotic stress resistance of the wheat genotypes (Additional file 2: Figure S2).

Two genes of *TaDREB1-D* existed simultaneously on D chromosome of all genotypes, named *TaDREB1-D1* amplified with the primer set *DREB1-D1F/R* and *TaDREB1-D2* amplified with the primer set *DREB1-D2F/R*. *TaDREB1-D1* and *TaDREB1-D2* had the similar nucleotide sequence except a 11 bp insertion in the former. In addition, two alleles of *TaDREB1-D1* were identified, named *TaDREB1-D11* and *TaDREB1-D12*, and also two alleles of *TaDREB1-D2* were identified, named *TaDREB1-D21* and *TaDREB1-D22*. *TaDREB1-D11* had the same sequence with *TaDREB1-D* (GenBank accession number: DQ195068.1), while eight SNPs were detected in *TaDREB1-D12*, of which 6 SNPs presented at position 1205 bp (G-A), 1264 bp (C-G), 1282 bp (A-G), 1340 bp (A-G), 1429 bp(C-A) and 1490 bp (A-G), respectively. Among the SNPs, three mutations changed the amino acids from A (1.8) to A (4.4), H (− 3.2) to R (− 4.5) and A (1.8) to E (− 3.5), respectively. The hydrophilicity of amino acids changed was generally enhanced. Another 2 SNPs were detected in 3’UTR of *TaDREB1-D12*. Moreover, *TaDREB1-D21* had the same sequence with *TaDREB1-D11* (DQ195068.1) except a 11 bp deletion (CCCATGCGGCG) at position of 481 bp–491 bp in intron; And *TaDREB1-D22* had the same sequence with *TaDREB1-D12* except a 11 bp deletion (CCCATGCGGCG) at position of 481 bp–491 bp in intron and a 3 bp (ATT) insertion at positon 1645-1647 bp in 3 ‘UTR (Additional file 3: Figure S3). However, several SNPs were found between *TaDREB1-D21* and *TaDREB1-D22*, the same as between *TaDREB1-D11* and *TaDREB1-D12*.

### Expression characterization of *TaDREB1-A*, *TaDREB1-B*, and *TaDREB1-D* in two genotypes with different seed germination osmotic stress tolerances

**Fig. 3:**
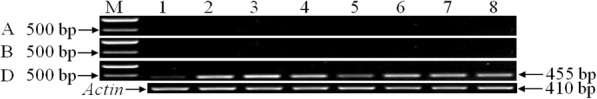
Expression of TaDREB1 Gene in Different osmotic stress Resistance and Different osmotic stress-treated Wheat Seeds by RT-PCR.M: DL-2000 DNA marker; Lanes 1–4: lines of 08–1783 (GDRI% = 55.98) (dry seeds treated with − 1.00 MPa mannitol for 0 h, 12 h, 24 h and 36 h); Lanes 5–8:variety of Zhangye 1 (GDRI% = 0.51)) (dry seeds treated with − 1.00 MPa mannitol for 0 h, 12 h, 24 h and 36 h).

In order to define the expression patterns of the three ***TaDREB1*** homologues and their relationship with osmotic stress tolerance, semi-quantitative RT-PCR analysis was carried out to determine the expression levels of ***TaDREB1-A***, ***TaDREB1-B***, and ***TaDREB1-D*** in two wheat genotypes differing in seed germination osmotic stress tolerance, using the *ACTIN* gene as an internal control. The results showed that the transcriptions of *TaDREB1-A* and *TaDREB1-B* genes were not detected in dry seeds and seeds treated with − 1.00 MPa mannitol for 12, 24 and 36 h via agarose gel electrophoresis. However, the expression level of *TaDREB1-D* had the tendency to increase gradually and then decrease when treated with − 1.00 MPa mannitol for 0 h, 12 h, 24 h and 36 h. Higher transcript expression level was detected in seeds treated by mannitol than in dry seeds. The highest transcript expression level came from osmotic stress-resistant line 08–1783 for 24 h treatment and from osmotic stress-sensitive variety Zhangye 1 after 12 h treatment (Fig. 3).

## Discussion

osmotic stress is the most significant environmental stress in agriculture worldwide, so the improvement of grain yield under water limitation is increasingly targeted in plant breeding program. Seed germination osmotic stress resistance is important for wheat cultivation, especially in seeding stage. *TaDREB* play an important role in response to drought stress [2]. Compared with the result studied by Wei et al. [11], much rich allelic variations were founded in our study. From the 6 genotypes tested, a total of 13 gene variations (*TaDREB1-A11*, *TaDREB1-A12*, *TaDREB1-A13*, *TaDREB1-A14*, *TaDREB1-A15*, *TaDREB1-B11*, *TaDREB1-B12*, *TaDREB1-B13*, *TaDREB1-B14*, *TaDREB1-D11*, *TaDREB1-D12*, *TaDREB1-D21 *and *TaDREB1-D22*) were identified, indicating that much richer allelic *TaDREB1*variations existed in common wheat. However, no relationship was found between the SNPs and GDRI for the time being.

In addition, two *TaDREB1-D* genes was detected in each of the 6 wheat genotypes tested, named *TaDREB1-D1* and *TaDREB1-D2*; And two alleles of *TaDREB1-D1* (*TaDREB1-D11* and *TaDREB1-D12*) and two alleles of *TaDREB1-D2* (*TaDREB1-D21* and *TaDREB1-D22*) were identified. It was interesting to find that *TaDREB1-D21* had the same sequence with *TaDREB1-D11* (DQ195068.1) except a 11 bp deletion (CCCATGCGGCG) at position of 481 bp–491 bp in intron; And *TaDREB1-D22* had the same sequence with *TaDREB1-D12* except a 11 bp deletion (CCCATGCGGCG) at position of 481 bp–491 bp in intron and a 3 bp (ATT) insertion at positon 1645-1647 bp in 3 ‘UTR (Additional file 3: Figure S3). However, several SNPs were found between *TaDREB1-D21* and *TaDREB1-D22*, the same as between *TaDREB1-D11* and *TaDREB1-D12*. The base similarity between *TaDREB1-D11* and *TaDREB1-D21* (or between *TaDREB1-D12* and *TaDREB1-D22*) was higher than between the two alleles of *TaDREB1-D1* (or between the two alleles of *TaDREB1-D2*). Besides, *TaDREB1-D11* and *TaDREB1-D21* always appeared together in the same genotype, so as for *TaDREB1-D12* and *TaDREB1-D22*. The results indicated that the duplication of *TaDREB1-D* gene occurred after the SNP mutations, and the copy of *TaDREB1-D11* and *TaDREB1-D12* (*TaDREB1-D21* and *TaDREB1-D22*) was independent in different genotypes or environments.

Higher transcript expression level of *TaDREB1-D* presented in seeds treated with mannitol, but no transcript product detected in *TaDREB1-A* and *TaDREB1-B*, the results showed that *TaDREB1-D* was more important to seed germination under osmotic stress than *TaDREB1-A* and *TaDREB1-B*. There were some reports that some genes homologies to DREB1 were weakly induced by osmotic stress [10, 19, 31], so same resulit were founded in *TaDREB1-A* and *TaDREB1-B* because of no transcript product detected in agrose gel. In addition, according to the complicated function of DREB in *Arabidopsis thaliana*, rice, soybean, cotton and other plants [18–23] Bohnert et al. 1995 and the transcript expression character of *TaDREB1-D*, more work needs to clarify the function of *TaDREB1-D* in further experiment.

When seeds were treated with − 1.00 MPa mannitol, the time of the highest transcript expression level of *TaDREB1-D* presented at 24 h in the line with osmotic stress resistance, but the highest transcript expression level of *TaDREB1-D* presented at 12 h in the variety with osmotic stress sensitivity. If the time that the highest transcript expression level of *TaDREB1-D* appeare in more genotypes with different osmotic stress tolerance and relate with seed germonation tolerance under osmotic stress, it could be used as a potential marker to identify the genotypes with more seed germination drought resistance in breeding program, and then to breed the stable and high yield varieties in dry farming conditions. It is necessary to characterize genetic resources based on osmotic stress adaptation, determine suitable genotypes, and use them to improve the wheat resistance. In the germplasms tested, 7 genotypes belong to level 1 osmotic stress tolerance were identified, (08–1783, 07–5866, 08–1826, 07–6239, Bayou 2, 34–206 and Linyou 2), which can be used as the parents in wheat breeding program for seed germination osmotic stress resistance.

## Conclusions

In summy, our study was intended to identify the seed germination resistance varieties, search the new allelic variation of *TaDREB1*, and character the transcript expression level of *TaDREB1-A*, *TaDREB1-B* and *TaDREB1-D* in wheat varieties or lines with different values of GDRI.

The results indicated that seven genotypes belong to the level 1 osmotic stress tolerance, which might be used in wheat breeding for drought resistence; thirteen new allele variations of ***TaDREB1*** were identified in the germplasms tested, in addition, *TaDREB1-D1* and *TaDREB1-D2* existed in each variety; In wheat seeds under osmotic stress, no transcript expression level of *TaDREB1-A* and *TaDREB1-B* were detected, but that of *TaDREB1-D*, and the capacity of osmotic stress tolerance was closely correlated with the expression level and tendency of *TaDREB1-D*.

## Additional files

Additional file 1: **Figure S1.** Sequence comparison of the *TaDREB1-A11*, *TaDREB1-A12*,*TaDREB1-A13*,*TaDREB1-A14*,*TaDREB1-A1*5 and *TaDREB1-A* (DQ195070 .1), SNPs shown with boldface letters. (DOCX 40 kb)

Additional file 2: **Figure S2.** Sequence comparison of the *TaDREB1-B11*, *TaDREB1-B12*,*TaDREB1-B13*,*TaDREB1-B14* and *TaDREB1-B* (DQ195069 .1), SNPs shown with boldface letters. (DOCX 19 kb)

Additional file 3: **Figure S3.** Sequence comparison of the *TaDREB1-D11*, *TaDREB1-D12*,*TaDREB1-D21*,*TaDREB1-D22* and *TaDREB1-D* (DQ195068.1), insertions are shadowed, SNPs shown with boldface letters. (DOCX 23 kb)

## Abbreviations

ABF: ABRE binding factor
AREB: ABA responsive element binding protein
CBF: C-repeat binding factor
CRT: C repeat
CTAB: Hexadecyltrimethylammonium bromide
DREB: Dehydration-responsive element binding
GDRI: Germination drought resistance index
UTR: Untranslated regions
