# Supplementary material for: Characterization of TaDREB1 in wheat genotypes with different seed germination under osmotic stress
Source: Hereditas. 2018 Aug 1;155:26. doi: 10.1186/s41065-018-0064-6 (PMC6090928; doi:10.1186/s41065-018-0064-6)
Supplement: Supplementary file 1 — Figure S1. Sequence comparison of the TaDREB1-A11, TaDREB1-A12,TaDREB1-A13,TaDREB1-A14,TaDREB1-A15 and TaDREB1-A (DQ195070 .1), SNPs shown with boldface letters. (DOCX 40 kb) [file 41065_2018_64_MOESM1_ESM.docx]

*TaDREB1-A11* CGGAACCACTCCCTCCATCTC**T**AC**T**T**G**TATCCCGCCGCG**.**TTTCGCTCGA 50

*TaDREB1-A12* CGGAACCACTCCCTCCATCTC**T**AC**T**T**G**TATCCCGCCGCG**.**TTTCGCTCGA 50

*TaDREB1-A13* CGGAACCACTCCCTCCATCTC**T**AC**T**T**G**TATCCCGCCGCG**.**TTTCGCTCGA 50

*TaDREB1-A14* CGGAACCACTCCCTCCATCTC**T**AC**T**T**G**TATCCCGCCGCG**.**TTTCGCTCGA 50

*TaDREB1-A15* CGGAACCACTCCCTCCATCTC**T**AC**T**T**G**TATCCCGCCGCG**.**TTTCGCTCGA 50

DQ195070.1       CGGAACCACTCCCTCCATCTCCACCTCTATCCCGCCGCGTTTTCGCTCGA    50

*TaDREB1-A11* ATCTACGGGCATTGCTGAGGCGGCGGCGGCGCGCCGTCGCCGCGACATGG    100

*TaDREB1-A12* ATCTACGGGCA TTGCTGAGGCGGCGGCGGCGCGCCGTCGCCGCGACATGG 100

*TaDREB1-A13* ATCTACGGGCA TTGCTGAGGCGGCGGCGGCGCGCCGTCGCCGCGACATGG 100

*TaDREB1-A14* ATCTACGGGCA TTGCTGAGGCGGCGGCGGCGCGCC**A**TCGCCGCGACATGG 100

*TaDREB1-A15* ATCTACGGGCATTGCTGAGGCGGCGGCGGCGCGCCGTCGCCGCGACATGG  100

DQ195070.1  ATCTACGGGCATTGCTGAGGCGGCGGCGGCGCGCCGTCGCCGCGACATGG    100

*TaDREB1-A11* AATTTGCGCGTGTTTCCGCGGTTG**G**TCGGT**G**C**T**GTGAAACATGCCCATCTA 151

*TaDREB1-A12* AATTTGCGCGTGTTTCCGCGGTTG**G**TCGGT**G**C**T**GTGAAACATGCCCATCTA 151

*TaDREB1-A13* AATTTGCGCGTGTTTCCGCGGTTG**G**TCGGT**G**C**T**GTGAAACATGCCCATCTA 151

*TaDREB1-A14* AATTTGCGCGTGTTTCCGCGGTTGCTCGGTACCGTGAAACATGCCCATCTA 151

*TaDREB1-A15* AATTTGCGCGTGTTTCCGCGGTTG**G**TCGGT**G**C**T**GTGAAACATGCCCATCTA 151

DQ195070.1  AATTTGCGCGTGTTTCCGCGGTTGCTCGGTACCGTGAAACATGCCCATCTA 151

*TaDREB1-A11* AAG**C**AG**T**TATCCGCT**AC**TCGTTTCCTGTTACCGCGGCGCGTGTCGGACTAG 202

*TaDREB1-A12* TAAG**C**AG**T**TATCCGCT**AC**TCGTTTCCTGTTACGCGGCGCGTGTCGGACTAG 202

*TaDREB1-A13* TAAG**C**AG**T**TATCCGCT**AC**TCGTTTCCTGTTACGCGGCGCGTGTCGGACTAG 202

*TaDREB1-A14* TAAGTAGCTATCCGCTGTTCGTTTCCTGTTACGCGGCGCGTGTCGGACTAG 202

*TaDREB1-A15* TAAG**C**AG**T**TATCCGCT**AC**TCGTTTCCTGTTACGCGGCGCGTGTCGGACTAG 202

DQ195070.1  TAAGTAGCTATCCGCTGTTCGTTTCCTGTTACGCGGCGCGTGTCGGACTAG 202

*TaDREB1-A11* TCGTAGGTCGGCGGTGGTCGCCCTAGGTCG**AT**GGC**T**G**T**CCT**C**CAGTTCGGT 253

*TaDREB1-A12* TCGTAGGTCGGCGGTGGTCGCCCTAGGTCG**AT**GGC**T**G**T**CCT**C**CAGTTCGGT 253

*TaDREB1-A13* TCGTAGGTCGGCGGTGGTCGCCCTAGGTCG**AT**GGC**T**G**T**CCT**C**CAGTTCGGT 253

*TaDREB1-A14* TCGTAGGTCGGCGGTGGTCGCCCTAGGTCGGCGGCCGCCCTTCAGTTCGGT   253

*TaDREB1-A15* TCGTAGGTCGGCGGTGGTCGCCCTAGGTCG**AT**GGC**T**G**T**CCT**C**CAGTTCGGT 253

DQ195070.1  TCGTAGGTCGGCGGTGGTCGCCCTAGGTCGGCGGCCGCCCTTCAGTTCGGT  253

*TaDREB1-A11*  TCGCCGGGTTTCCGCCGGCT**GT**AGAATTATGGTTTCCTCCG**T**CTTCTCGGTT 305

*TaDREB1-A12* TCGCCGGGTTTCCGCCGGCT**GT**AGAATTATGGTTTCCTCCG**T**CTTCTCGGTT 305

*TaDREB1-A13* TCGCCGGGTTTCCGCCGGCT**GT**AGAATTATGGTTTCCTCCG**T**CTTCTCGGTT 305

*TaDREB1-A14* TCGCCGGGTTTCCGCCGGCTACAGAATTATGGTTTCCTCCG**T**CTTCTCGGTT 305

*TaDREB1-A15* TCGCCGGGTTTCCGCCGGCT**GT**AGAATTATGGTTTCCTCCG**T**CTTCTCGGTT 305

DQ195070.1  TCGCCGGGTTTCCGCCGGCTACAGAATTATGGTTTCCTCCGCCTTCTCGGTT   305

*TaDREB1-A11* GGGGA**T**TGGAGTTCGGCGAGATTCAGAGCGG**T**GCGTGGCGAACTGTCGGT  355

*TaDREB1-A12* GGGGA**T**TGGAGTTCGGCGAGATTCAGAGCGG**T**GCGTGGCGAACTGTCGGT  355

*TaDREB1-A13* GGGGA**T**TGGAGTTCGGCGAGATTCAGAGCGG**T**GCGTGGCGAACTGTCGGT  355

*TaDREB1-A14* G**A**GGA**T**TGGAGTTCGGCGAGATTCAGAGCGG**T**GCGTGGCGAACTGTCGGT 355

*TaDREB1-A15* GGGGA**T**TGGAGTTCGGCGAGATTCAGAGCGG**T**GCGTGGCGAACTGTCGGT 355

DQ195070.1  GGGGACTGGAGTTCGGCGAGATTCAGAGCGGCGCGTGGCGAACTGTCGGT 355

*TaDREB1-A11* GTT**G**GG TCGGGTGAGGCGGCGCCGTCCTCTGAATCGGATGCAGAAGTCCC 405

*TaDREB1-A12* GTT**G**GGTCGGGTGAGGCGGCGCCGTCCTCTGAATCGGATGCAGAAGTCCC 405

*TaDREB1-A13* GTT**G**GGTCGGGTGAGGCGGCGCCGTCCTCTGAATCGGATGCAGAAGTCCC 405

*TaDREB1-A14* GTT**G**GGTCGGGTGAGGCGGCGCCGTCCTCTGAATCGGATGCAGAAGTCCC 405

*TaDREB1-A15* GTT**G**GGTCGGGTGAGGCGGCGCCGTCCTCTGAATCGGATGCAGAAGTCCC 405

DQ195070.1  GTTAGGTCGGGTGAGGCGGCGCCGTCCTCTGAATCGGATGCAGAAGTCCC 405

*TaDREB1-A11* CATGTCCATGGCGGCGGTCGGGGATTGCCCATGCCATGGCGGTACGA**..** TTG 457

*TaDREB1-A12* CATGTCCATGGCGGCGGTCGGGGATTGCCCATGCCATGGCGGTACGA**..**TTG 457

*TaDREB1-A13* CA**C**GTCCATGGCGGCGGTCGGGGATTGCCCATGCCATGGCGGTACGA**..**TTG  457

*TaDREB1-A14* CATGTCCATGGCGGCGGTCGGGGATTGCCCATGCCATGGCGGTACGA**TT**TTG 457

*TaDREB1-A15* CATGTCCATGGCGGCGGTCGGGGATTGCCCATGCCATGGCGGTACGA**..**TTG 457

DQ195070.1  CATGTCCATGGCGGCGGTCGGGGATTGCCCATGCCATGGCGGTACGATTTTG 457

*TaDREB1-A11*  CTGGTGCA**C**AGCATCATAAACTATATC**T**TCGGG**A**GCTCGATTGCGGTTGGTA 509

*TaDREB1-A12* CTGGTGCA**C**AGCATCATAAACTATATC**T**TCGGG**A**GCTCGATTGCGGTTGGTA 509

*TaDREB1-A13* CTGGTGCA**C**AGCATCATAAACTATATC**T**TCGGG**A**GCTCGATTGCGGTTGGTA 509

*TaDREB1-A14* CTGGTGCATAGCATCATAAACTATATCCTCGGGGGCTCGATTGCGGTTGGTA 509

*TaDREB1-A15* CTGGTGCA**C**AGCATCATAAACTATATC**T**TCGGG**A**GCTCGATTGCGGTTGGTA 509

DQ195070.1  CTGGTGCATAGCATCATAAACTATATCCTCGGGGGCTCGATTGCGGTTGGTA 509

*TaDREB1-A11* CCCAACCCAAGTGATAATAATCTCCTTGAC**T**TTTTTCCACCAAGGAAACAAG 561

*TaDREB1-A12* CCCAACCCAAGTGATAATAATCTCCTTGAC**T**TTTTTCCACCAAGGAAACAAG  561

*TaDREB1-A13* CCCAACCCAAGTGATAATAATCTCCTTGAC**T**TTTTTCCACCAAGGAAACAAG  561

*TaDREB1-A14* CCCAACCCAAGTGATAATAATCTCCTTGAC.TTTTTCCACCAAGGAAACAAG 561

*TaDREB1-A15* CCCAACCCAAGTGATAATAATCTCCTTGAC**T**TTTTTCCACCAAGGAAACAAG  561

DQ195070.1  CCCAACCCAAGTGATAATAATCTCCTTGAC**.** TTTTTCCACCAAGGAAACAAG 561

*TaDREB1-A11* GATAGCCCTGCTTCGTT**T**TGTTTTAGATTTATACGACTTTTTTTT**TCTG**TGAGA 615

*TaDREB1-A12* GATAGCCCTGCTTCGTT**T**TGTTTTAGATTTATACGACTTTTTTTT**TCTG**TGAGA 615

*TaDREB1-A13* GATAGCCCTGCTTCGTT**T**TGTTTTAGATTTATACGACTTTTTTTT**TCTG**TGAGA 615

*TaDREB1-A14* GATAGCCCTGCTTCGTTCTGTTTTAGATTTATACGACTTTTTTTT**A** . . . TGAGA 615

*TaDREB1-A15* GATAGCCCTGCTTCGTT**T**TGTTTTAGATTTATACGACTTTTTTTT**TCTG**TGAGA 615

DQ195070.1  GATAGCCCTGCTTCGTTCTGTTTTAGATTTATACGACTTTTTTTT**A** . . . TGAGA 615

*TaDREB1-A11* AAGATT**C**ATATGA**CT**CTG**A**CTG**C**TTATGTTTTT**G**GTTTCAA**C**GTGTTTTCA**C**CT 669

*TaDREB1-A12* AAGATT**C**ATATGA**CT**CTG**A**CTG**C**TTATGTTTTT**G**GTTTCAA**C**GTGTTTTCA**C**CT 669

*TaDREB1-A13* AAGATT**C**ATATGA**CT**CTG**A**CTG**C**TTATGTTTTT**G**GTTTCAA**C**GTGTTTTCA**C**CT 669

*TaDREB1-A14* AAGATTTATATGA . . CTGGCTGGTTATGTTTTTTGTTTCAAAGTGTTTTCA. CT 669

*TaDREB1-A15* AAGATTCATATGA**CT**CTG**A**CTG**C**TTATGTTTTT**G**GTTTCAA**C**GTGTTTTCA**C**CT 669

DQ195070 .1 AAGATTTATATGA . . CTGGCTGGTTATGTTTTTTGTTTCAAAGTGTTTTCA . CT  669

*TaDREB1-A11*  TGTGATATGGATTGCCTTGATGAACAGGAAGAAGAAAGTGCGCAGGAGAAG 720

*TaDREB1-A12*  TGTGATATGGATTGCCTTGATGAACAGGAAGAAGAAAGTGCGCAGGAGAAG 720

*TaDREB1-A13* TGTGATATGGATTGCCTTGATGAACAGGAAGAAGAAAGTGCGCAGGAGAAG 720

*TaDREB1-A14* TGTGATATGGATTGCCTTGATGAACAGGAAGAAGAAAGTGCGCAGGAGAAG 720

*TaDREB1-A15*  TGTGATATGGATTGCCTTGATGAACAGGAAGAAGAAAGTGCGCAGGAGAAG 720

DQ195070.1  TGTGATATGGATTGCCTTGATGAACAGGAAGAAGAAAGTGCGCAGGAGAAG 720

*TaDREB1-A11* CAC**T**GGTCCTGATTCGGTTGCTGAAACCATCAAGAAGTGGAAGGAGGAAAA 771

*TaDREB1-A12* CAC**T**GGTCCTGATTCGGTTGCTGAAACCATCAAGAAGTGGAAGGAGGAAAA 771

*TaDREB1-A13* CAC**T**GGTCCTGATTCGGTTGCTGAAACCATCAAGAAGTGGAAGGAGGAAAA 771

*TaDREB1-A14* CACCGGTCCTGATTCGGTTGCTGAAACCATCAAGAAGTGGAAGGAGGAAAA 771

*TaDREB1-A15* CAC**T**GGTCCTGATTCGGTTGCTGAAACCATCAAGAAGTGGAAGGAGGAAAA 771

DQ195070.1  CACCGGTCCTGATTCGGTTGCTGAAACCATCAAGAAGTGGAAGGAGGAAAA 771

*TaDREB1-A11* CCAGAAGCTCCAGCAAGAGAATGGATCCCGGAAAGCACCGGCCAAGGGTTC 822

*TaDREB1-A12* CCAGAAGCTCCAGCAAGAGAATGGATCCCGGAAAGCACCGGCCAAGGGTTC 822

*TaDREB1-A13* CCAGAAGCTCCAGCAAGAGAATGGATCCCGGAAAGCACCGGCCAAGGGTTC 822

*TaDREB1-A14* CCAGAAGCTCCAGCAAGAGAATGGATCCCGGAAAGCACCGGCCAAGGGTTC 822

*TaDREB1-A15* CCAGAAGCTCCAGCAAGAGAATGGATCCCGGAAAGCACCGGCCAAGGGTTC 822

DQ195070.1  CCAGAAGCTCCAGCAAGAGAATGGATCCCGGAAAGCACCGGCCAAGGGTTC 822

*TaDREB1-A11* CAAGAAAGGGTGCATGGCAGGGAAAGGAGGTCCAGAGAATTCAAACTGCGC 873

*TaDREB1-A12* CAAGAAAGGGTGCATGGCAGGGAAAGGAGGTCCAGAGAATTCAAACTGCGC 873

*TaDREB1-A13* C AAGAAAGGGTGCATGGCAGGGAAAGGAGGTCCAGAGAATTCAAACTGCGC 873

*TaDREB1-A14* C AAGAAAGGGTGCATGGCAGGGAAAGGAGGTCCAGAGAATTCAAACTGCGC 873

*TaDREB1-A15* C AAGAAAGGGTGCATGGCAGGGAAAGGAGGTCCAGAGAATTCAAACTGCGC 873

DQ195070 .1 CAAGAAAGGGTGCATGGCAGGGAAAGGAGGTCCAGAGAATTCAAACTGCGC 873

*TaDREB1-A11* TTACCGCGGTGTGAGGCAGAGGACGTGGGGGAAATGGGTTGCTGAGATCCGT 925

*TaDREB1-A12* TTACCGCGGTGTGAGGCAGAGGACGTGGGGGAAATGGGTTGCTGAGATCCGT 925

*TaDREB1-A13* TTACCGCGGTGTGAGGCAGAGGACGTGGGGGAAATGGGTTGCTGAGATCCGT 925

*TaDREB1-A14* TTACCGCGGTGTGAGGCAGAGGACGTGGGGGAAATGGGTTGCTGAGATCCGT 925

*TaDREB1-A15* TTACCGCGGTGTGAGGCAGAGGACGTGGGGGAAATGGGTTGCTGAGATCCGT 925

DQ195070 .1 TTACCGCGGTGTGAGGCAGAGGACGTGGGGGAAATGGGTTGCTGAGATCCGT 925

*TaDREB1-A11* GAGCCCAACCGTGGCAATCGGCTGTGGCTTGGTTCATTCCCTAC**C**GCAGTCG A   978

*TaDREB1-A12* GAGCCCAACCGTG**A**CAATCGGCTGTGGCTTGGTTCATTCCCTAC**C**GCAGTCGA 978

*TaDREB1-A13* GAGCCCAACCGTGGCAATCGGCTGTGGCTTGGTTCATTCCCTAC**C**GCAGTCGA 978

*TaDREB1-A14* GAGCCCAACCGTGGCAATCGGCTGTGGCTTGGTTCATTCCCTACTGCAGTCGA 978

*TaDREB1-A15* GAGCCCAACCGTGGCAATCGGCTGTGGCTTGGTTCATTCCCTAC**C**GCAGTCGA 978

DQ195070 .1 GAGCCCAACCGTGGCAATCGGCTGTGGCTTGGTTCATTCCCTACTGCAGTCGA 978

*TaDREB1-A11* AGCTGCACGTGCATATGATGATGCGGCAAGGGCAATGTATGGCGCCAAAGCAC 1031

*TaDREB1-A12* AGCTGCACGTGCATATGATGATGCGGCAAGGGCAATGTATGGCGCCAAAGCAC 1031

*TaDREB1-A13* AGCTGCACGTGCATATGATGATGCGGCAAGGGCAATGTATGGCGCCAAAGCAC 1031

*TaDREB1-A14* AGCTGCACGTGCATATGATGATGCGGCAAGGGCAATGTATGGCGCCAAAGCAC 1031

*TaDREB1-A15* AGCTGC**G**CGTGCATATGATGATGCGGCAAGGGCAATGTATGGCGCCAAAGCAC 1031

DQ195070 .1 AGCTGCACGTGCATATGATGATGCGGCAAGGGCAATGTATGGCGCCAAAGCAC 1031

*TaDREB1-A11* GTGTCAACTTCTCAGAGCAGTCCCCGGATGCCAACTCTGGTTGCACGCTGGCA 1084

*TaDREB1-A12* GTGTCAACTTCTCAGAGCAGTCCCCGGATGCCAACTCTGGTTGCACGCTGGCA 1084

*TaDREB1-A13* GTGTCAACTTCTCAGAGCAGTCCCCGGATGCCAACTCTGGTTGCACGCTGGCA 1084

*TaDREB1-A14* GTGTCAACTTCTCAGAGCAGTCCCCGGATGCCAACTCTGGTTGCACGCTGGCA 1084

*TaDREB1-A15* G*TG*TCAACTTCTCAGAGCAGTCCCCGGATGCCAACTCTGGTTGCACGCTGGCA 1084

DQ195070 .1 G*TG*TCAACTTCTCAGAGCAGTCCCCGGATGCCAACTCTGGTTGCACGCTGGCA 1084

*TaDREB1-A11* CCTCCATTGCTGACGTCTAATGGGGCAACCGCTGCATCACATCCTTCTGATGGG 1138

*TaDREB1-A12* CCTCCATTGCTGACGTCTAATGGGGCAACCGCTGCATCACATCCTTCTGATGGG 1138

*TaDREB1-A13* CCTCCATTGCTGACGTCTAATGGGGCAACCGCTGCATCACATCCTTCTGATGGG 1138

*TaDREB1-A14* CCTCCATTGCTGACGTCTAATGGGGCAACCGCTGCATCACATCCTTCTGATGGG 1138

*TaDREB1-A15* CCTCCATTGCTGACGTCTAATGGGGCAACCGCTGCATCACATCCTTCTGATGGG 1138

DQ195070 .1 CCTCCATTGCTGACGTCTAATGGGGCAACCGCTGCATCACATCCTTCTGATGGG 1138

*TaDREB1-A11* AAGGATGAATCGGAGTCTCCTCCTTCTCTTATCTCAAATGGGCCGACAGCTGCG 1192

*TaDREB1-A12* AAGGATGAATCGGAGTCTCCTCCTTCTCTTATCTCAAATGGGCCGACAGCTGCG 1192

*TaDREB1-A13* AAGGATGAATCGGAGTCTCCTCCTTCTCTTATCTCAAATGGGCCGACAGCTGCG 1192

*TaDREB1-A14* AAGGATGAATCGGAGTCTCCTCCTTCTCTTATCTCAAATGGGCCGACAGCTGCG 1192

*TaDREB1-A15* AAGGATGAATCGGAGTCTCCTCCTTCTCTTATCTCAAATGGGCCGACAGCTGCG 1192

DQ195070 .1 AAGGATGAATCGGAGTCTCCTCCTTCTCTTATCTCAAATGGGCCGACAGCTGCG 1192

*TaDREB1-A11* CTGCGTCGGTCTGATGCTAAGGATGAGTCTGAGTCTGCAGGGACCGTGGCACG 1245

*TaDREB1-A12* CTGCGTCGGTCTGATGCTAAGGATGAGTCTGAGTCTGCAGGGACCGTGGCACG 1245

*TaDREB1-A13* CTGCGTCGGTCTGATGCTAAGGATGAGTCTGAGTCTGCAGGGACCGTGGCACG 1245

*TaDREB1-A14*  CTGCGTCGGTCTGATGCTAAGGATGAGTCTGAGTCTGCAGGGACCGTGGCACG 1245

*TaDREB1-A1*5 CTGCGTCGGTCTGATGCTAAGGATGAGTCTGAGTCTGCAGGGACCGTGGCACG 1245

DQ195070 .1 CTGCGTCGGTCTGATGCTAAGGATGAGTCTGAGTCTGCAGGGACCGTGGCACG 1245

*TaDREB1-A11* TAAGGTGAAGAAAGAAGTGAGCAATGATTTGAGAAGTACCCATGAGGAGCAC 1297

*TaDREB1-A12* TAAGGTGAAGAAAGAAGTGAGCAATGATTTGAGAAGTACCCATGAGGAGCAC 1297

*TaDREB1-A13* TAAGGTGAAGAAAGAAGTGAGCAATGATTTGAGAAGTACCCATGAGGAGCAC 1297

*TaDREB1-A14* TAAGGTGAAGAAAGAAGTGAGCAATGATTTGAGAAGTACCCATGAGGAGCAC 1297

*TaDREB1-A1*5 TAAGGTGAAGAAAGAAGTGAGCAATGATTTGAGAAGTACCCATGAGGAGCAC 1297

DQ195070 .1 TAAGGTGAAGAAAGAAGTGAGCAATGATTTGAGAAGTACCCATGAGGAGCAC 1297

*TaDREB1-A11* AAGACCCTGGAAGTATCCCAACCAAAAGGGAAGGCTTTACATAAAGAAGCGA 1349

*TaDREB1-A12* AAGACCCTGGAAGTATCCCAACCAAAAGGGAAGGCTTTACATAAAGAAGCGA 1349

*TaDREB1-A13* AAGACCCTGGAAGTATCCCAACCAAAAGGGAAGGCTTTACATAAAGAAGCGA 1349

*TaDREB1-A14* AAGACCCTGGAAGTATCCCAACCAAAAGGGAAGGCTTTACATAAAGAAGCGA 1349

*TaDREB1-A1*5 AAGACCCTGGAAGTATCCCAACCAAAAGGGAAGGCTTTACATAAAGAAGCGA 1349

DQ195070 .1 AAGACCCTGGAAGTATCCCAACCAAAAGGGAAGGCTTTACATAAAGAAGCGA 1349

*TaDREB1-A11* ACGTAAGTTATGATTACTTCAACGTCGAGGAAGTTCTTGACATGATAATTGTGG 1403

*TaDREB1-A12* ACGTAAGTTATGATTACTTCAACGTCGAGGAAGTTCTTGACATGATAATTGTGG 1403

*TaDREB1-A13* ACGTAAGTTATGATTACTTCAACGTCGAGGAAGTTCTTGACATGATAATTGTGG 1403

*TaDREB1-A14* ACGTAAGTTATGATTACTTCAACGTCGAGGAAGTTCTTGACATGATAATTGTGG 1403

*TaDREB1-A1*5 ACGTAAGTTATGATTACTTCAACGTCGAGGAAGTTCTTGACATGATAATTGTGG 1403

DQ195070 .1 ACGTAAGTTATGATTACTTCAACGTCGAGGAAGTTCTTGACATGATAATTGTGG 1403

*TaDREB1-A11* AGTTGAGTGCTGATGTAAAAATGGAAGCACATGAAGAGTACCAAGATGGTGAT 1456

*TaDREB1-A12* AGTTGAGTGCTGATGTAAAAATGGAAGCACATGAAGAGTACCAAGATGGTGAT 1456

*TaDREB1-A13* AGTTGAGTGCTGATGTAAAAATGGAAGCACATGAAGAGTACCAAGATGGTGAT 1456

*TaDREB1-A14* AGTTGAGTGCTGATGTAAAAATGGAAGCACATGAAGAGTACCAAGATGGTGAT 1456

*TaDREB1-A1*5 AGTTGAGTGCTGATGTAAAAATGGAAGCACATGAAGAGTACCAAGATGGTGAT 1456

DQ195070 .1 AGTTGAGTGCTGATGTAAAAATGGAAGCACATGAAGAGTACCAAGATGGTGAT 1456

*TaDREB1-A11* GATGGGTTTAGTCTTTTCTCATATTAGGGTTTTAGCTATGAGGGTTGTAGTCATG 1511

*TaDREB1-A12* GATGGGTTTAGTCTTTTCTCATATTAGGGTTTTAGCTATGAGGGTTGTAGTCATG 1511

*TaDREB1-A13* GATGGGTTTAGTCTTTTCTCATATTAGGGTTTTAGCTATGAGGGTTGTAGTCATG 1511

*TaDREB1-A14* GATGGGTTTAGTCTTTTCTCATATTAGGGTTTTAGCTATGAGGGTTGTAGTCATG 1511

*TaDREB1-A15* GATGGGTTTAGTCTTTTCTCATATTAGGGTTTTAGCTATGAGGGTTGTAGTCATG 1511

DQ195070 .1 GATGGGTTTAGTCTTTTCTCATATTAGGGTTTTAGCTATGAGGGTTGTAGTCATG 1511

*TaDREB1-A11* CGGAGCAATAGGGATAACTTTCATTCTAGCTGCTAGGAAATACTTCAAATTATC 1565

*TaDREB1-A12* CGGAGCAATAGGGATAACTTTCATTCTAGCTGCTAGGAAATACTTCAAATTATC 1565

*TaDREB1-A13* CGGAGCAATAGGGATAACTTTCATTCTAGCTGCTAGGAAATACTTCAAATTATC 1565

*TaDREB1-A14* CGGAGCAATAGGGATAACTTTCATTCTAGCTGCTAGGAAATACTTCAAATTATC 1565

*TaDREB1-A1*5 CGGAGCAATAGGGATAACTTTCATTCTAGCTGCTAGGAAATACTTCAAATTATC 1565

DQ195070 .1 CGGAGCAATAGGGATAACTTTCATTCTAGCTGCTAGGAAATACTTCAAATTATC 1565

*TaDREB1-A11* TGCAACCCGAAGCTCTGTAGTCACTTATGGTTTTCATCTTACTGGAGAGAATA 1618

*TaDREB1-A12* TGCAACCCGAAGCTCTGTAGTCACTTATGGTTTTCATCTTACTGGAGAGAATA 1618

*TaDREB1-A13* TGCAACCCGAAGCTCTGTAGTCACTTATGGTTTTCATCTTACTGGAGAGAATA 1618

*TaDREB1-A14* TGCAACCCGAAGCTCTGTAGTCACTTATGGTTTTCATCTTACTGGAGAGAATA 1618

*TaDREB1-A15* TGCAACCCGAAGCTCTGTAGTCACTTATGGTTTTCATCTTACTGGAGAGAATA 1618

DQ195070 .1 TGCAACCCGAAGCTCTGTAGTCACTTATGGTTTTCATCTTACTGGAGAGAATA 1618

*TaDREB1-A11* GCTTTATACCATAAGTCAACGGGTACAAGAAGTTGTCCTGTGCGTTGAGTTCA 1671

*TaDREB1-A12* GCTTTATACCATAAGTCAACGGGTACAAGAAGTTGTCCTGTGCGTTGAGTTCA 1671

*TaDREB1-A13* GCTTTATACCATAAGTCAACGGGTACAAGAAGTTGTCCTGTGCGTTGAGTTCA 1671

*TaDREB1-A14* GCTTTATACCATAAGTCAACGGGTACAAGAAGTTGTCCTGTGCGTTGAGTTCA 1671

*TaDREB1-A15* GCTTTATACCATAAGTCAACGGGTACAAGAAGTTGTCCTGTGCGTTGAGTTCA 1671

DQ195070 .1 GCTTTATACCATAAGTCAACGGGTACAAGAAGTTGTCCTGTGCGTTGAGTTCA 1671

*TaDREB1-A11* TGTACT 1677

*TaDREB1-A12* TGTACT 1677

*TaDREB1-A13* TGTACT 1677

*TaDREB1-A14* TGTACT 1677

*TaDREB1-A1*5 TGTACT 1677

DQ195070 .1 TGTACT 1677

Fig.S1 Sequence comparison of the *TaDREB1-A11*, *TaDREB1-A12*，*TaDREB1-A13*，*TaDREB1-A14*，*TaDREB1-A1*5 and *TaDREB1-A* (DQ195070 .1 ), SNPs shown with boldface letters.
