# Supplementary material for: Characterization of TaDREB1 in wheat genotypes with different seed germination under osmotic stress
Source: Hereditas. 2018 Aug 1;155:26. doi: 10.1186/s41065-018-0064-6 (PMC6090928; doi:10.1186/s41065-018-0064-6)
Supplement: Supplementary file 2 — Figure S2. Sequence comparison of the TaDREB1-B11, TaDREB1-B12,TaDREB1-B13,TaDREB1-B14 and TaDREB1-B (DQ195069 .1), SNPs shown with boldface letters. (DOCX 19 kb) [file 41065_2018_64_MOESM2_ESM.docx]

*TaDREB1-B11* CCCAACCCAAGTGATAATAATCTCCTTGACTTTTTTCCACCAAGGAAACA 50

*TaDREB1-B11* CCCAACCCAAGTGATAATAATCTCCTTGACTTTTTTCCACCAAGGAAACA 50

*TaDREB1-B13* CCCAACCCAAGTGATAATAATCTCCTTGACTTTTTTCCACCAAGGAAACA 50

*TaDREB1-B14* CCCAACCCAAGTGATAATAATCTCCTTGACTTTTTTCCACCAAGGAAACA 50

DQ195069 .1 CCCAACCCAAGTGATAATAATCTCCTTGACTTTTTTCCACCAAGGAAACA 50

*TaDREB1-B11* AGGATAGCCCTGCTTTGGTTTGTTTTAGATTTATAC**A**ACTTTTTTTTCTCTG 102

*TaDREB1-B12* AGGATAGCC**T**TGCTTTGGTTTGTTTTAGATTTATACGACTTTTTTTTCTCTG 102

*TaDREB1-B13* AGGATAGCC**T**TGCTTTGGTTTGTTTTAGATTTATACGACTTTTTTTTCTCTG 102

*TaDREB1-B14* AGGATAGCCCTGCTTTGGTTTGTTTTAGATTTATAC**A**ACTTTTTTTTCTCTG 102

DQ195069 .1 AGGATAGCCCTGCTTTGGTTTGTTTTAGATTTATACGACTTTTTTTTCTCTG 102

*TaDREB1-B11* AGAAAGATTTATATGACTCTGACTGCTTATGTTTTTTGTTTCAACGTGGTT 153

*TaDREB1-B12* AGAAAGATTTATATGACTCTGACTGCTTATGTTTTTTGTTTCA**G**CGTGGTT 153

*TaDREB1-B13*  AGAAAGATTTATATGACTCTGACTGCTTATGTTTTTTGTTTCAACGTGGTT 153

*TaDREB1-B14* AGAAAGATTTATATGACTCTGACTGCTTATGTTTTTTGTTTCAACGTGGTT 153

DQ195069 .1 AGAAAGATTTATATGACTCTGACTGCTTATGTTTTTTGTTTCAACGTGGTT 153

*TaDREB1-B11* TCACCTTGTGATATGGATTGCCTTGATGA**A**CAGGAAGAAGAAAGTGCGC 202

*TaDREB1-B12* TCACCTTGTGATATGGATTGCCTTGATGACC**T**G**C**AAGAAGAAAGTGCGC 202

*TaDREB1-B13*  TCACCTTGTGATATGGATTGCCTTGATGACCAGGAAGA AGAAAGTGCGC 202

*TaDREB1-B14*  TCACCTTGTGATATGGATTGCCTTGATGA**A**CAGGAAGA AGAAAGTGCGC 202

DQ195069 .1 TCACCTTGTGATATGGATTGCCTTGATGACCAGGAAGAAGAAAGTGCGC 202

*TaDREB1-B11* AGGAGAAGCACTGGTCCTGATTCGGTTGCTGAAACCATCAAGAAGTGGA 251

*TaDREB1-B12*  A**T**GAGAAGCACTGGTCC**G**GATTC**A**GTTGCTGAAACCATCAAGAAGTGGA 251

*TaDREB1-B13* AGGAGAAGCACTGGTCCTGATTCGGTTGCTGAAACCATCAAGAAGTGGA 251

*TaDREB1-B14* AGGAGAAGCACTGGTCCTGATTCGGTTGCTGAAACCATCAAGAAGTGGA 251

DQ195069 .1 AGGAGAAGCACTGGTCCTGATTCGGTTGCTGAAACCATCAAGAAGTGGA 251

*TaDREB1-B11*  AGGAGGAAAACCAGAAGCTCCTGCAAGAGAATGGATCCCGGAAAGCACC 300

*TaDREB1-B12* AGGAGGAAA**G**CC**G**GAAGCTCCTGCAAGAGAATGGATCCCGGAAAGCACC 300

*TaDREB1-B13*  *A*GGAGGAAAACCAGAAGCTCCTGCAAGAGAATGGATCCCGGAAAGCACC 300

*TaDREB1-B14* AGGAGGAAAACCAGAAGCTCCTGCAAGAGAATGGA**C**CCCGGAAAGCACC  300

DQ195069 .1 AGGAGGAAAACCAGAAGCTCCTGCAAGAGAATGGATCCCGGAAAGCACC 300

*TaDREB1-B11* GGCCAAGGGTTCCAAGAAAGGGTGCATGGCAGGGAAAGGAGGTCCAGAG 349

*TaDREB1-B12* GGCCAAGGGTTCCAAGAAAGGGTGCATGGCAGGGAAAGGAGGTCCAGAG 349

*TaDREB1-B13* GGCCAAGGGTTCCAAGAAAGGGTGCATGGCAGGGAAAGGAGGTCCAGAG 349

*TaDREB1-B14* GGCCAAGGGTTCCAAGAAAGGGTGCATGGCAGGGAAAGGAGGTCCAGAG 349

DQ195069 .1 GGCCAAGGGTTCCAAGAAAGGGTGCATGGCAGGGAAAGGAGGTCCAGAG 349

*TaDREB1-B11* AATTCAAACTGCGCTTACCGCGGTGTGAGGCAGAGGACGTGGGGCAAATG 399

*TaDREB1-B12* AATTCAAACTGCGCTTACCGCGGTGTGAGGCAGA**A**GAC**C**TGGGGCAAATG  399

*TaDREB1-B13* AATTCAAACTGCGCTTACCGCGGTGTGAGGCAGAGGACGTGGGGCAAATG 399

*TaDREB1-B14* AATTCAAACTGCGCTTACCGCGGTGTGAGGCAGAGGACGTGGGGCAAATG 399

DQ195069 .1 AATTCAAACTGCGCTTACCGCGGTGTGAGGCAGAGGACGTGGGGCAAATG 399

*TaDREB1-B11* GGTTGCTGAGATCCGTGAGCCCAACCGTGGCAACCGGCTGTGGCT**C**GGTT 449

*TaDREB1-B12* GGTTGCTGA**A**ATCCGTGAGCCCAACCGTGGCAACCGGCTGTGGCTTGGTT 449

*TaDREB1-B13* GGTTGCTGAGATCCGTGAGCCCAACCGTGGCAACCGGCTGTGGCTTGGTT 449

*TaDREB1-B14* GGTTGCTGAGATCCGTGAGCCCAACCGTGGCAACCGGCTGTGGCTTGGTT 449

DQ195069 .1 GGTTGCTGAGATCCGTGAGCCCAACCGTGGCAACCGGCTGTGGCTTGGTT 449

*TaDREB1-B11* CATTCCCTACCGCAGTCGAAGCTGCACGTGCATATGATGATGCGGCAAGGG 500

*TaDREB1-B12* CATTCCCTACCGCAGTCGAAGCT**ATC**CGTGCATATGATGATGCGGCAAGGG  500

*TaDREB1-B13* CATTCCCTACCGCAGTCGAAGCTGCACGTGCATATGATGATGCGGCAAGGG 500

*TaDREB1-B14* CATTCCCTACCGCAGTCGAAGCTGCACGTGCATATGATGATGCGGCAAGGG 500

DQ195069 .1 CATTCCCTACCGCAGTCGAAGCTGCACGTGCATATGATGATGCGGCAAGGG 500

*TaDREB1-B11*  CA**G**TGTATGGCG**T**CAAAGCACGTGTCAACTTCTCAGAGCAGTCCCCAGATG     551

*TaDREB1-B12* CAATGTATGGCGCC**T**AAGCACGTGTCAACTTCTCA**A**A**T**CAGTCCCCAGATG  551

*TaDREB1-B13* CAATGTATGGCGCCAAAGCACGTGTCAACTTCTCAGAGCAGTCCCCAGATG 551

*TaDREB1-B14* CAATGTATGGCGCCAAAGCACGTGTCAACTTCTCAGAGCAGTCCCCAGATG 551

DQ195069 .1 CAATGTATGGCGCCAAAGCACGTGTCAACTTCTCAGAGCAGTCCCCAGATG 551

*TaDREB1-B11* CCAGCTCTGGTTGCACGCTGGCACCTCCATTGCTGATGTCTAATGGGGCA**G**  602

*TaDREB1-B12* CCAGCTCTGGTTGCAC**C**CTGGC**C**CCTCCATTGCTGATGTCTAATGGGGCAA  602

*TaDREB1-B13* CCAGCTCTGGTTGCACGCTGGCACCTCCATTGCTGATGTCTAATGGGGCAA 602

*TaDREB1-B14* CCAGCTCTGGTTGCACGCTGGCACCTCCATTGCTGATGTCTAATGGGGCAA 602

DQ195069 .1 CCAGCTCTGGTTGCACGCTGGCACCTCCATTGCTGATGTCTAATGGGGCAA 602

*TaDREB1-B11* CTGCCGCATCACATCCTTCTGATGGGAAGGATGAGTCTGAGTCTGCAGGGA 653

*TaDREB1-B12* CTGCCGCATCACATCCTTCTGATGGGAAGGATGAGTCTGA**T**TCTGCA**A**GGA 653

*TaDREB1-B13* CTGCCGCATCACATCCTTCTGATGGGAAGGATGAGTCTGAGTCTGCAGGGA 653

*TaDREB1-B14* CTGCCGCATCACATCCTTCTGATGGGAAGGATGAGTCTGAGTCTGCAGGGA 653

DQ195069 .1 CTGCCGCATCACATCCTTCTGATGGGAAGGATGAGTCTGAGTCTGCAGGGA 653

*TaDREB1-B11* CCGTGGCACATAAGGTG.AAAAAAGAAGTGAGCAATGA 691

*TaDREB1-B12*  **T**CGTG**C**CACATAA**C**GTGAAAAAAAGAA**T**TGAGCAATGA 691

*TaDREB1-B13* CCGTGGCACATAAGGTG.AAAAAAGAAGTGAGCAATGA 691

*TaDREB1-B14* CCGTGGCACATAAGGTG.AAAAAAGAAGTGAGCAATGA 691

DQ195069 .1 CCGTGGCACATAAGGTG.AAAAAAGAAGTGAGCAATGA 691

Fig.S2 Sequence comparison of the *TaDREB1-B11*, *TaDREB1-B12*，*TaDREB1-B13*，*TaDREB1-B14* and *TaDREB1-B* (DQ195069 .1 ), SNPs shown with boldface letters.
