# Supplementary material for: Characterization of TaDREB1 in wheat genotypes with different seed germination under osmotic stress
Source: Hereditas. 2018 Aug 1;155:26. doi: 10.1186/s41065-018-0064-6 (PMC6090928; doi:10.1186/s41065-018-0064-6)
Supplement: Supplementary file 3 — Figure S3. Sequence comparison of the TaDREB1-D11, TaDREB1-D12,TaDREB1-D21,TaDREB1-D22 and TaDREB1-D (DQ195068.1), insertions are shadowed, SNPs shown with boldface letters. (DOCX 23 kb) [file 41065_2018_64_MOESM3_ESM.docx]

*TaDREB1-D11* TCGTCCCTCTTCTCGCTCCATGGAGACCGGGGGTAGCAAGCGGGAAGGAG 50

*TaDREB1-D12* TCGTCCCTCTTCTCGCTCCATGGAGACCGGGGGTAGCAAGCGGGAAGGAG 50

*TaDREB1-D21* TCGTCCCTCTTCTCGCTCCATGGAGACCGGGGGTAGCAAGCGGGAAGGAG 50

*TaDREB1*-D22 TCGTCCCTCTTCTCGCTCCATGGAGACCGGGGGTAGCAAGCGGGAAGGAG 50

DQ195068.1 TCGTCCCTCTTCTCGCTCCATGGAGACCGGGGGTAGCAAGCGGGAAGGAG 50

*TaDREB1*-D11 ACTGCCCCGGGCAGGAAAGGTGAGCGCCGGAACCACTCCCTCCATCTCTA 100

*TaDREB1*-D12 ACTGCCCCGGGCAGGAAAGGTGAGCGCCGGAACCACTCCCTCCATCTCTA 100

*TaDREB1*-D21 ACTGCCCCGGGCAGGAAAGGTGAGCGCCGGAACCACTCCCTCCATCTCTA 100

*TaDREB1*-D22 ACTGCCCCGGGCAGGAAAGGTGAGCGCCGGAACCACTCCCTCCATCTCTA 100

DQ195068.1 ACTGCCCCGGGCAGGAAAGGTGAGCGCCGGAACCACTCCCTCCATCTCTA 100

*TaDREB1*-D11 CTTGTATCCCGCCGCGTTTCGCTCGAATCTACGGGCATTGCTGAGGCGGC 150

*TaDREB1*-D12 CTTGTATCCCGCCGCGTTTCGCTCGAATCTACGGGCATTGCTGAGGCGGC 150

*TaDREB1*-D21 CTTGTATCCCGCCGCGTTTCGCTCGAATCTACGGGCATTGCTGAGGCGGC 150

TaDREB1-D22 CTTGTATCCCGCCGCGTTTCGCTCGAATCTACGGGCATTGCTGAGGCGGC 150

DQ195068.1 CTTGTATCCCGCCGCGTTTCGCTCGAATCTACGGGCATTGCTGAGGCGGC 150

*TaDREB1-D11* GGCGGCGCGCCGTCGCCGCGACATGGAATTTGCGCGTGTTTCCGCGGTTG 200

*TaDREB1-D12* GGCGGCGCGCCGTCGCCGCGACATGGAATTTGCGCGTGTTTCCGCGGTTG 200

*TaDREB1-D21* GGCGGCGCGCCGTCGCCGCGACATGGAATTTGCGCGTGTTTCCGCGGTTG 200

*TaDREB1*-D22 GGCGGCGCGCCGTCGCCGCGACATGGAATTTGCGCGTGTTTCCGCGGTTG 200

DQ195068.1 GGCGGCGCGCCGTCGCCGCGACATGGAATTTGCGCGTGTTTCCGCGGTTG 200

*TaDREB1-D11* GTCGGTGCTGTGAAACATGCCCATCTAAAGCAGTTATCCGCTACTCGTTT 250

*TaDREB1*-D12 GTCGGTGCTGTGAAACATGCCCATCTAAAGCAGTTATCCGCTACTCGTTT 250

*TaDREB1*-D21 GTCGGTGCTGTGAAACATGCCCATCTAAAGCAGTTATCCGCTACTCGTTT 250

*TaDREB1*-D22 GTCGGTGCTGTGAAACATGCCCATCTAAAGCAGTTATCCGCTACTCGTTT 250

DQ195068.1 GTCGGTGCTGTGAAACATGCCCATCTAAAGCAGTTATCCGCTACTCGTTT 250

*TaDREB1*-D11 CCTGTTACCGCGGCGCGTGTCGGACTAGTCGTAGGTCGGCGGTGGTCGCC 300

*TaDREB1*-D12 CCTGTTACCGCGGCGCGTGTCGGACTAGTCGTAGGTCGGCGGTGGTCGCC 300

*TaDREB1-D21* CCTGTTACCGCGGCGCGTGTCGGACTAGTCGTAGGTCGGCGGTGGTCGCC 300

*TaDREB1-D22* CCTGTTACCGCGGCGCGTGTCGGACTAGTCGTAGGTCGGCGGTGGTCGCC 300

DQ195068.1 CCTGTTACCGCGGCGCGTGTCGGACTAGTCGTAGGTCGGCGGTGGTCGCC 300

*TaDREB1-D11* CTAGGTCGATGGCTGTCCTCCAGTTCGGTTCGCCGGGTTTCCGCCGGCTG 350

*TaDREB1*-D12 CTAGGTCGATGGCTGTCCTCCAGTTCGGTTCGCCGGGTTTCCGCCGGCTG 350

*TaDREB1*-D21 CTAGGTCGATGGCTGTCCTCCAGTTCGGTTCGCCGGGTTTCCGCCGGCTG 350

*TaDREB1*-D22 CTAGGTCGATGGCTGTCCTCCAGTTCGGTTCGCCGGGTTTCCGCCGGCTG 350

DQ195068.1 CTAGGTCGATGGCTGTCCTCCAGTTCGGTTCGCCGGGTTTCCGCCGGCTG 350

*TaDREB1-D11* TAGAATTATGGTTTCCTCCGTCTTCTCGGTTGGGGATTGGAGTTCGGCGA 400

*TaDREB1-D12* TAGAATTATGGTTTCCTCCGTCTTCTCGGTTGGGGATTGGAGTTCGGCGA 400

*TaDREB1-D21* TAGAATTATGGTTTCCTCCGTCTTCTCGGTTGGGGATTGGAGTTCGGCGA 400

*TaDREB1-D22* TAGAATTATGGTTTCCTCCGTCTTCTCGGTTGGGGATTGGAGTTCGGCGA 400

DQ195068.1 TAGAATTATGGTTTCCTCCGTCTTCTCGGTTGGGGATTGGAGTTCGGCGA 400

*TaDREB1-D11* GATTCAGAGCGGTGCGTGGCGAACTGTCGGTGTTGGGTCGGGTGAGGCGG 450

*TaDREB1-D12*  GATTCAGAGCGGTGCGTGGCGAACTGTCGGTGTTGGGTCGGGTGAGGCGG 450

*TaDREB1-D21* GATTCAGAGCGGTGCGTGGCGAACTGTCGGTGTTGGGTCGGGTGAGGCGG 450

*TaDREB1-D22* GATTCAGAGCGGTGCGTGGCGAACTGTCGGTGTTGGGTCGGGTGAGGCGG 450

DQ195068.1 GATTCAGAGCGGTGCGTGGCGAACTGTCGGTGTTGGGTCGGGTGAGGCGG 450

*TaDREB1-D11* CGCCGTCCTCTGAATCGGATGCAGAAGTCCCC**...........**ATGTCCA 489

*TaDREB1-D12* CGCCGTCCTCTGAATCGGATGCAGAAGTCCCC**...........**ATGTCCA 489

*TaDREB1-D21* CGCCGTCCTCTGAATCGGATGCAGAAGTCCCC**CATGCGGCGGC**ATGTCCA 500

*TaDREB1-D22* CGCCGTCCTCTGAATCGGATGCAGAAGTCCCC**CATGCGGCGGC**ATGTCCA 500

DQ195068.1 CGCCGTCCTCTGAATCGGATGCAGAAGTCCCC**...........**ATGTCCA 489

*TaDREB1-D11* TGGCGGCGGTCGGGGATTGCCCATGCCATGGCGGTACGATTGCTGGTGCA 539

*TaDREB1-D12* TGGCGGCGGTCGGGGATTGCCCATGCCATGGCGGTACGATTGCTGGTGCA 539

*TaDREB1-D21* TGGCGGCGGTCGGGGATTGCCCATGCCATGGCGGTACGATTGCTGGTGCA 550

*TaDREB1-D22* TGGCGGCGGTCGGGGATTGCCCATGCCATGGCGGTACGATTGCTGGTGCA 550

DQ195068.1 TGGCGGCGGTCGGGGATTGCCCATGCCATGGCGGTACGATTGCTGGTGCA 539

*TaDREB1-D11* CAGCATCATAAACTATATCTTCGGGAGCTCGATTGCGGTTGGTACCCAAC 589

*TaDREB1-D12*  CAGCATCATAAACTATATCTTCGGGAGCTCGATTGCGGTTGGTACCCAAC 589

*TaDREB1-D21*  CAGCATCATAAACTATATCTTCGGGAGCTCGATTGCGGTTGGTACCCAAC 600

*TaDREB1-D22*  CAGCATCATAAACTATATCTTCGGGAGCTCGATTGCGGTTGGTACCCAAC 600

DQ195068.1 CAGCATCATAAACTATATCTTCGGGAGCTCGATTGCGGTTGGTACCCAAC 589

*TaDREB1-D11* CCAAGTGATAATAATCTCCTTGACTTTTTTCCACCAAGGAAACAAGGATA 639

*TaDREB1-D12* CCAAGTGATAATAATCTCCTTGACTTTTTTCCACCAAGGAAACAAGGATA 639

*TaDREB1-D21*  CCAAGTGATAATAATCTCCTTGACTTTTTTCCACCAAGGAAACAAGGATA 650

*TaDREB1-D22* CCAAGTGATAATAATCTCCTTGACTTTTTTCCACCAAGGAAACAAGGATA 650

DQ195068.1 CCAAGTGATAATAATCTCCTTGACTTTTTTCCACCAAGGAAACAAGGATA 639

*TaDREB1-D11* GCCCTGCTTCGTTTTGTTTTAGATTTATACGACTTTTTTTTTCTGTGAGA 689

*TaDREB1-D12* GCCCTGCTTCGTTTTGTTTTAGATTTATACGACTTTTTTTTTCTGTGAGA 689

*TaDREB1-D21* GCCCTGCTTCGTTTTGTTTTAGATTTATACGACTTTTTTTTTCTGTGAGA 700

*TaDREB1-D22* GCCCTGCTTCGTTTTGTTTTAGATTTATACGACTTTTTTTTTCTGTGAGA 700

DQ195068.1 GCCCTGCTTCGTTTTGTTTTAGATTTATACGACTTTTTTTTTCTGTGAGA 689

*TaDREB1-D11* AAGATTCATATGACTCTGACTGCTTATGTTTTTGGTTTCAACGTGTTTTC 739

*TaDREB1-D12* AAGATTCATATGACTCTGACTGCTTATGTTTTTGGTTTCAACGTGTTTTC 739

*TaDREB1-D21*  AAGATTCATATGACTCTGACTGCTTATGTTTTTGGTTTCAACGTGTTTTC 750

*TaDREB1-D22* AAGATTCATATGACTCTGACTGCTTATGTTTTTGGTTTCAACGTGTTTTC 750

DQ195068.1 AAGATTCATATGACTCTGACTGCTTATGTTTTTGGTTTCAACGTGTTTTC 739

*TaDREB1-D11* ACCTTGTGATATGGATTGCCTTGATGAACAGGAAGAAGAAAGTGCGCAGG 789

*TaDREB1-D12*  ACCTTGTGATATGGATTGCCTTGATGAACAGGAAGAAGAAAGTGCGCAGG 789

*TaDREB1-D21*  ACCTTGTGATATGGATTGCCTTGATGAACAGGAAGAAGAAAGTGCGCAGG 800

*TaDREB1-D22*  ACCTTGTGATATGGATTGCCTTGATGAACAGGAAGAAGAAAGTGCGCAGG 800

DQ195068.1 ACCTTGTGATATGGATTGCCTTGATGAACAGGAAGAAGAAAGTGCGCAGG 789

*TaDREB1-D11* AGAAGCACTGGTCCTGATTCGGTTGCTGAAACCATCAAGAAGTGGAAGGA 839

*TaDREB1-D12* AGAAGCACTGGTCCTGATTCGGTTGCTGAAACCATCAAGAAGTGGAAGGA 839

*TaDREB1-D21* AGAAGCACTGGTCCTGATTCGGTTGCTGAAACCATCAAGAAGTGGAAGGA 850

*TaDREB1-D22* AGAAGCACTGGTCCTGATTCGGTTGCTGAAACCATCAAGAAGTGGAAGGA 850

DQ195068.1 AGAAGCACTGGTCCTGATTCGGTTGCTGAAACCATCAAGAAGTGGAAGGA 839

*TaDREB1-D11* GGAAAACCAGAAGCTCCAGCAAGAGAATGGATCCCGGAAAGCACCGGCCA 889

*TaDREB1-D12* GGAAAACCAGAAGCTCCAGCAAGAGAATGGATCCCGGAAAGCACCGGCCA 889

*TaDREB1-D21* GGAAAACCAGAAGCTCCAGCAAGAGAATGGATCCCGGAAAGCACCGGCCA 900

*TaDREB1-D22* GGAAAACCAGAAGCTCCAGCAAGAGAATGGATCCCGGAAAGCACCGGCCA 900

DQ195068.1 GGAAAACCAGAAGCTCCAGCAAGAGAATGGATCCCGGAAAGCACCGGCCA 889

*TaDREB1-D11* AGGGTTCCAAGAAAGGGTGCATGGCAGGGAAAGGAGGTCCAGAGAATTCA 939

*TaDREB1-D12* AGGGTTCCAAGAAAGGGTGCATGGCAGGGAAAGGAGGTCCAGAGAATTCA 939

*TaDREB1-D21*  AGGGTTCCAAGAAAGGGTGCATGGCAGGGAAAGGAGGTCCAGAGAATTCA 950

*TaDREB1-D22* AGGGTTCCAAGAAAGGGTGCATGGCAGGGAAAGGAGGTCCAGAGAATTCA 950

DQ195068.1 AGGGTTCCAAGAAAGGGTGCATGGCAGGGAAAGGAGGTCCAGAGAATTCA 939

*TaDREB1-D11* AACTGCGCTTACCGCGGTGTGAGGCAGAGGACGTGGGGGAAATGGGTTGC 989

*TaDREB1-D12*  AACTGCGCTTACCGCGGTGTGAGGCAGAGGACGTGGGGGAAATGGGTTGC 989

*TaDREB1-D21* AACTGCGCTTACCGCGGTGTGAGGCAGAGGACGTGGGGGAAATGGGTTGC 1000

*TaDREB1-D22*  AACTGCGCTTACCGCGGTGTGAGGCAGAGGACGTGGGGGAAATGGGTTGC 1000

DQ195068.1 AACTGCGCTTACCGCGGTGTGAGGCAGAGGACGTGGGGGAAATGGGTTGC 989

*TaDREB1-D11* TGAGATCCGTGAGCCCAACCGTGGCAATCGGCTGTGGCTTGGTTCATTCC 1039

*TaDREB1-D12*  TGAGATCCGTGAGCCCAACCGTGGCAATCGGCTGTGGCTTGGTTCATTCC 1039

*TaDREB1-D21* TGAGATCCGTGAGCCCAACCGTGGCAATCGGCTGTGGCTTGGTTCATTCC 1050

*TaDREB1-D22*  TGAGATCCGTGAGCCCAACCGTGGCAATCGGCTGTGGCTTGGTTCATTCC 1050

DQ195068.1 TGAGATCCGTGAGCCCAACCGTGGCAATCGGCTGTGGCTTGGTTCATTCC 1039

*TaDREB1-D11* CTACCGCAGTCGAAGCTGCACGTGCATATGATGATGCGGCAAGGGCAATG 1089

*TaDREB1-D12* CTACCGCAGTCGAAGCTGCACGTGCATATGATGATGCGGCAAGGGCAATG 1089

*TaDREB1-D21*  CTACCGCAGTCGAAGCTGCACGTGCATATGATGATGCGGCAAGGGCAATG 1100

*TaDREB1-D22*  CTACCGCAGTCGAAGCTGCACGTGCATATGATGATGCGGCAAGGGCAATG 1100

DQ195068.1 CTACCGCAGTCGAAGCTGCACGTGCATATGATGATGCGGCAAGGGCAATG 1089

*TaDREB1-D11* TATGGCGCCAAAGCACGTGTCAACTTCTCAGAGCAGTCCCCGGATGCCAA 1139

*TaDREB1-D12*  TATGGCGCCAAAGCACGTGTCAACTTCTCAGAGCAGTCCCCGGATGCCAA 1139

*TaDREB1-D21* TATGGCGCCAAAGCACGTGTCAACTTCTCAGAGCAGTCCCCGGATGCCAA 1150

*TaDREB1-D22* TATGGCGCCAAAGCACGTGTCAACTTCTCAGAGCAGTCCCCGGATGCCAA 1150

DQ195068.1 TATGGCGCCAAAGCACGTGTCAACTTCTCAGAGCAGTCCCCGGATGCCAA 1139

*TaDREB1-D11* CTCTGGTTGCACGCTGGCACCTCCATTGCCGATGTCTAATGGGGCAACCG 1189

*TaDREB1-D12* CTCTGGTTGCACGCTGGCACCTCCATTGCCGATGTCTAATGGGGCAACCG 1189

*TaDREB1-D21*  CTCTGGTTGCACGCTGGCACCTCCATTGCCGATGTCTAATGGGGCAACCG 1200

*TaDREB1-D22*  CTCTGGTTGCACGCTGGCACCTCCATTGCCGATGTCTAATGGGGCAACCG 1200

DQ195068.1 CTCTGGTTGCACGCTGGCACCTCCATTGCCGATGTCTAATGGGGCAACCG 1189

*TaDREB1-D11* CTGCGTCACATCCTTCTGATGGGAAGGATGAATCGGAGTCTCCTCCTTCT 1239

*TaDREB1-D12* CTGC**A**TCACATCCTTCTGATGGGAAGGATGAATCGGAGTCTCCTCCTTCT 1239

*TaDREB1-D21*  CTGCGTCACATCCTTCTGATGGGAAGGATGAATCGGAGTCTCCTCCTTCT 1250

*TaDREB1-D22* CTGC**A**TCACATCCTTCTGATGGGAAGGATGAATCGGAGTCTCCTCCTTCT 1250

DQ195068.1 CTGCGTCACATCCTTCTGATGGGAAGGATGAATCGGAGTCTCCTCCTTCT 1239

*TaDREB1-D11* CTTATCTCAAATGCGCCGACAGCTGCGCTGCATCGGTCTGATGCTAAGGA 1289

*TaDREB1-D12*  CTTATCTCAAATG**G**GCCGACAGCTGCGCTGC**G**TCGGTCTGATGCTAAGGA 1289

*TaDREB1-D21*  CTTATCTCAAATGCGCCGACAGCTGCGCTGCATCGGTCTGATGCTAAGGA 1300

*TaDREB1-D22*  CTTATCTCAAATG**G**GCCGACAGCTGCGCTGC**G**TCGGTCTGATGCTAAGGA 1300

DQ195068.1 CTTATCTCAAATGCGCCGACAGCTGCGCTGCATCGGTCTGATGCTAAGGA 1289

*TaDREB1-D11* TGAGTCTGAGTCTGCAGGGACCGTGGCACGTAAGGTGAAAAAAGAAGTGA 1339

*TaDREB1-D12* TGAGTCTGAGTCTGCAGGGACCGTGGCACGTAAGGTGAA**G**AAAGAAGTGA 1339

*TaDREB1-D21* TGAGTCTGAGTCTGCAGGGACCGTGGCACGTAAGGTGAAAAAAGAAGTGA 1350

*TaDREB1-D22* GAGTCTGAGTCTGCAGGGACCGTGGCACGTAAGGTGAA**G**AAAGAAGTGA 1350

DQ195068.1 TGAGTCTGAGTCTGCAGGGACCGTGGCACGTAAGGTGAAAAAAGAAGTGA 1339

*TaDREB1-D11* GCAATGATTTGAGAAGTACCCATGAGGAGCACAAGACCCTGGAAGTATCC 1389

*TaDREB1-D12* GCAATGATTTGAGAAGTACCCATGAGGAGCACAAGACCCTGGAAGTATCC 1389

*TaDREB1-D21* GCAATGATTTGAGAAGTACCCATGAGGAGCACAAGACCCTGGAAGTATCC 1400

*TaDREB1-D22* GCAATGATTTGAGAAGTACCCATGAGGAGCACAAGACCCTGGAAGTATCC 1400

DQ195068.1 GCAATGATTTGAGAAGTACCCATGAGGAGCACAAGACCCTGGAAGTATCC 1389

*TaDREB1-D11* CAACCAAAAGGGAAGGCTTTACATAAAGCAGCGAACGTAAGTTATGATTA 1439

*TaDREB1-D12*  CAACCAAAAGGGAAGGCTTTACATAAAG**A**AGCGAACGTAAGTTATGATTA 1439

*TaDREB1-D21* CAACCAAAAGGGAAGGCTTTACATAAAGCAGCGAACGTAAGTTATGATTA 1450

*TaDREB1-D22* CAACCAAAAGGGAAGGCTTTACATAAAG**A**AGCGAACGTAAGTTATGATTA 1450

DQ195068.1 CAACCAAAAGGGAAGGCTTTACATAAAGCAGCGAACGTAAGTTATGATTA 1439

*TaDREB1-D11* CTTCAACGTCGAGGAAGTTCTTGACATGATAATTGTGGAATTGAGTGCTG 1489

*TaDREB1-D12* CTTCAACGTCGAGGAAGTTCTTGACATGATAATTGTGGA**G**TTGAGTGCTG 1489

*TaDREB1-D21*  CTTCAACGTCGAGGAAGTTCTTGACATGATAATTGTGGAATTGAGTGCTG 1500

*TaDREB1-D22*  CTTCAACGTCGAGGAAGTTCTTGACATGATAATTGTGGA**G**TTGAGTGCTG 1500

DQ195068.1 CTTCAACGTCGAGGAAGTTCTTGACATGATAATTGTGGAATTGAGTGCTG 1489

*TaDREB1-D11* ATGTAAAAATGGAAGCACATGAAGAGTACCAAGATGGTGATGATGGGTTT 1539

*TaDREB1-D12* ATGTAAAAATGGAAGCACATGAAGAGTACCAAGATGGTGATGATGGGTTT 1539

*TaDREB1-D21*  ATGTAAAAATGGAAGCACATGAAGAGTACCAAGATGGTGATGATGGGTTT 1550

*TaDREB1-D22* ATGTAAAAATGGAAGCACATGAAGAGTACCAAGATGGTGATGATGGGTTT 1550

DQ195068.1 ATGTAAAAATGGAAGCACATGAAGAGTACCAAGATGGTGATGATGGGTTT 1539

*TaDREB1-D11* AGTCTTTTCTCATATTAGGGTTTTAGCTATGAGGGTTGCAGTCATGCGGA 1589

*TaDREB1-D12* AGTCTTTTCTCATATTAGGGTTTTAGCTATGAGGGTTG**T**AGTCATGCGGA 1589

*TaDREB1-D21*  AGTCTTTTCTCATATTAGGGTTTTAGCTATGAGGGTTGCAGTCATGCGGA 1600

*TaDREB1-D22* AGTCTTTTCTCATATTAGGGTTTTAGCTATGAGGGTTG**T**AGTCATGCGGA 1600

DQ195068.1 AGTCTTTTCTCATATTAGGGTTTTAGCTATGAGGGTTGCAGTCATGCGGA 1589

*TaDREB1-D11* GCAATAGGGATAACTTTCATTCTAGCTGCTAGGAAATACTTCAA**...**ATC 1636

*TaDREB1-D12*  GCAATAGGGATAACTTTCATTCTAGCTGCTAGGAAATACTTCAA**ATT**ATC 1639

*TaDREB1-D21* GCAATAGGGATAACTTTCATTCTAGCTGCTAGGAAATACTTCAA**...**ATC 1647

*TaDREB1-D22*  GCAATAGGGATAACTTTCATTCTAGCTGCTAGGAAATACTTCAA**ATT**ATC 1650

DQ195068.1 GCAATAGGGATAACTTTCATTCTAGCTGCTAGGAAATACTTCAA**...**ATC 1636

*TaDREB1-D11* TGCAACCCGAAGCTTTGTAGTCACTTATGGTTTTCATCTTACTGGAGAGA 1686

*TaDREB1-D12* TGCAACCCGAAGCT**C**TGTAGTCACTTATGGTTTTCATCTTACTGGAGAGA 1689

*TaDREB1-D21* TGCAACCCGAAGCTTTGTAGTCACTTATGGTTTTCATCTTACTGGAGAGA 1697

*TaDREB1-D22*  TGCAACCCGAAGCT**C**TGTAGTCACTTATGGTTTTCATCTTACTGGAGAGA 1700

DQ195068.1 TGCAACCCGAAGCTTTGTAGTCACTTATGGTTTTCATCTTACTGGAGAGA 1686

*TaDREB1-D11* ATAGCTTTATACCATAAGTCAACGGGTACAAGAAGTTGTCCTGTGCGTTG 1736

*TaDREB1-D12*  ATAGCTTTATACCATAAGTCAACGGGTACAAGAAGTTGTCCTGTGCGTTG 1739

*TaDREB1-D21* ATAGCTTTATACCATAAGTCAACGGGTACAAGAAGTTGTCCTGTGCGTTG 1747

*TaDREB1-D22*  ATAGCTTTATACCATAAGTCAACGGGTACAAGAAGTTGTCCTGTGCGTTG 1750

DQ195068.1 ATAGCTTTATACCATAAGTCAACGGGTACAAGAAGTTGTCCTGTGCGTTG 1736

*TaDREB1-D11* AGTTCATGTACT........... 1748

*TaDREB1-D12*  AGTTCATGTACT........... 1751

*TaDREB1-D21* AGTTCATGTACT........... 1759

*TaDREB1-D22*  AGTTCATGTACT........... 1762

DQ195068.1 AGTTCATGTACT........... 1748

Fig.S3 Sequence comparison of the *TaDREB1-D11*, *TaDREB1-D12*，*TaDREB1-D21*，*TaDREB1-D22* and *TaDREB1-D* (DQ195068.1 ), insertions are shadowed, SNPs shown with boldface letters.
